# β-Caryophyllene Acts as a Ferroptosis Inhibitor to Ameliorate Experimental Colitis

**DOI:** 10.3390/ijms232416055

**Published:** 2022-12-16

**Authors:** Yan-Ting Wu, Li-Shan Zhong, Chen Huang, Yu-Ying Guo, Fu-Jun Jin, Yu-Ze Hu, Zi-Bo Zhao, Zhe Ren, Yi-Fei Wang

**Affiliations:** 1 Department of Cell Biology, College of Life Science and Technology, Jinan University, Guangzhou 510632, China; 2 Guangdong Province Key Laboratory of Bioengineering Medicine, Jinan University, Guangzhou 510632, China; 3 Guangdong Provincial Biotechnology Drug & Engineering Technology Research Center, Jinan University, Guangzhou 510632, China; 4 National Engineering Research Center for Modernization of Traditional Chinese Medicine Moxa Herb Branch, Guangzhou 510632, China; 5 Key Laboratory of Innovative Technology Research on Natural Products and Cosmetics Raw Materials, Guangzhou 510632, China; 6 National Engineering and Technology Research Center of Modernization of Traditional Chinese Medicine Artemisia Argyi Branch Center, Guangzhou 510632, China

**Keywords:** ulcerative colitis, ferroptosis, β-caryophyllene, type 2 cannabinoid receptor, inflammation, macrophage

## Abstract

Macrophage infiltration is one of the main pathological features of ulcerative colitis (UC) and ferroptosis is a type of nonapoptotic cell death, connecting oxidative stress and inflammation. However, whether ferroptosis occurs in the colon macrophages of UC mice and whether targeting macrophage ferroptosis is an effective approach for UC treatment remain unclear. The present study revealed that macrophage lipid peroxidation was observed in the colon of UC mice. Subsequently, we screened several main components of essential oil from Artemisia argyi and found that β-caryophyllene (BCP) had a good inhibitory effect on macrophage lipid peroxidation. Additionally, ferroptotic macrophages were found to increase the mRNA expression of tumor necrosis factor alpha (Tnf-α) and prostaglandin-endoperoxide synthase 2 (Ptgs2), while BCP can reverse the effects of inflammation activated by ferroptosis. Further molecular mechanism studies revealed that BCP activated the type 2 cannabinoid receptor (CB2R) to inhibit macrophage ferroptosis and its induced inflammatory response both in vivo and in vitro. Taken together, BCP potentially ameliorated experimental colitis inflammation by inhibiting macrophage ferroptosis. These results revealed that macrophage ferroptosis is a potential therapeutic target for UC and identified a novel mechanism of BCP in ameliorating experimental colitis.

## 1. Introduction

Ulcerative colitis (UC) is a chronic relapsing colorectal inflammatory disease with low mortality. However, it is almost incurable. UC is mainly characterized by bleeding, diarrhea, relapsing and remitting mucosal inflammation of the colon and rectum [1]. As a chronic inflammatory disease, UC has a complex pathogenesis and is often closely related to diet, living environment, social pressure and numerous other factors. With the development of society, the incidence and prevalence of UC have been rising substantially all over the world [2]. The direct and indirect cost of UC is considerable [3]. Individuals with colitis not only have to spend substantial money and time on treatment, but also suffer from social pressure and lower quality of life, which hinders personal career development and impairs their mental health [4]. There are at least 27 novel drugs for UC with either recently completed or active trials [5]. However, the problems of drug tolerance and adverse events still remain. With the increase in patients with UC, the need for effective medicines of UC will be greater than ever, which will cause an increased burden on society [2,4]. Therefore, it is particularly important to find novel potential targets and novel effective therapeutic drugs with little toxicity or side effects.

Ferroptosis is a novel type of nonapoptotic cell death mainly characterized by iron overload, glutathione (GSH) exhaustion, extensive lipid peroxidation [6,7,8] and inflammatory reaction [9,10]. Studies have found that ferroptosis is not only closely related to cancer [11,12,13], but also involved in the occurrence and development of numerous inflammatory diseases, such as acute renal failure [14], acute kidney injury [15], steatohepatitis [16,17] and experimental autoimmune encephalomyelitis [18]. Notably, recent research has revealed that intestinal symptoms of dextran sulfate sodium (DSS)-induced colitis in mice were ameliorated by suppressing ferroptosis [19]. Furthermore, ferrostatin-1 (Fer-1), a ferroptosis inhibitor, effectively relieves DSS-induced colitis [20], which suggests that inhibiting ferroptosis may be a novel therapeutic strategy for UC. Although several studies have reported the association between ferroptosis and colitis, it remains unclear whether ferroptosis occurs in the colon macrophages of UC mice, and the relationship between ferroptosis and inflammation in UC is rarely reported.

β-Caryophyllene (BCP) is a bicyclic sesquiterpene type-flavoring and fragrance material, which widely exists in numerous plant essential oils [21]. The Standardization Administration Commission of the People’s Republic of China stipulates that BCP can be used as a natural flavoring for food (GB 2760-2014) [22], proving its safety. Furthermore, BCP has been reported to display effective antioxidant and anti-inflammatory activities through different mechanisms [23]. Increasing evidence from several studies has revealed that BCP has protective properties in a variety of inflammatory diseases, such as colitis [24,25], arthritis [26], alcoholic steatohepatitis [27], nonalcoholic steatohepatitis [28], multiple sclerosis [29] and neuropathic pain [30]. Although the wide pharmacological effects of BCP have been discovered, the effect of BCP on macrophage ferroptosis remains to be elucidated. The present study first investigated the occurrence of macrophage ferroptosis in UC mice. BCP was demonstrated to alleviate colitis by increasing glutathione peroxidase 4 (GPX4) expression, GPX activity and GSH content, reducing the levels of malondialdehyde (MDA) and 4-hydroxynonenal (4-HNE) in the colon of UC mice. Further experiments demonstrated that BCP suppressed macrophage ferroptosis by activating the type 2 cannabinoid receptor (CB2R). These results provided a novel perspective and potential for a pharmacological target for UC and identified that BCP acts as a ferroptosis inhibitor in vivo and in vitro.

## 2. Results

### 2.1. Ferroptosis Mediated the Pathogenesis of DSS-Induced UC Mice

**Figure 1:**
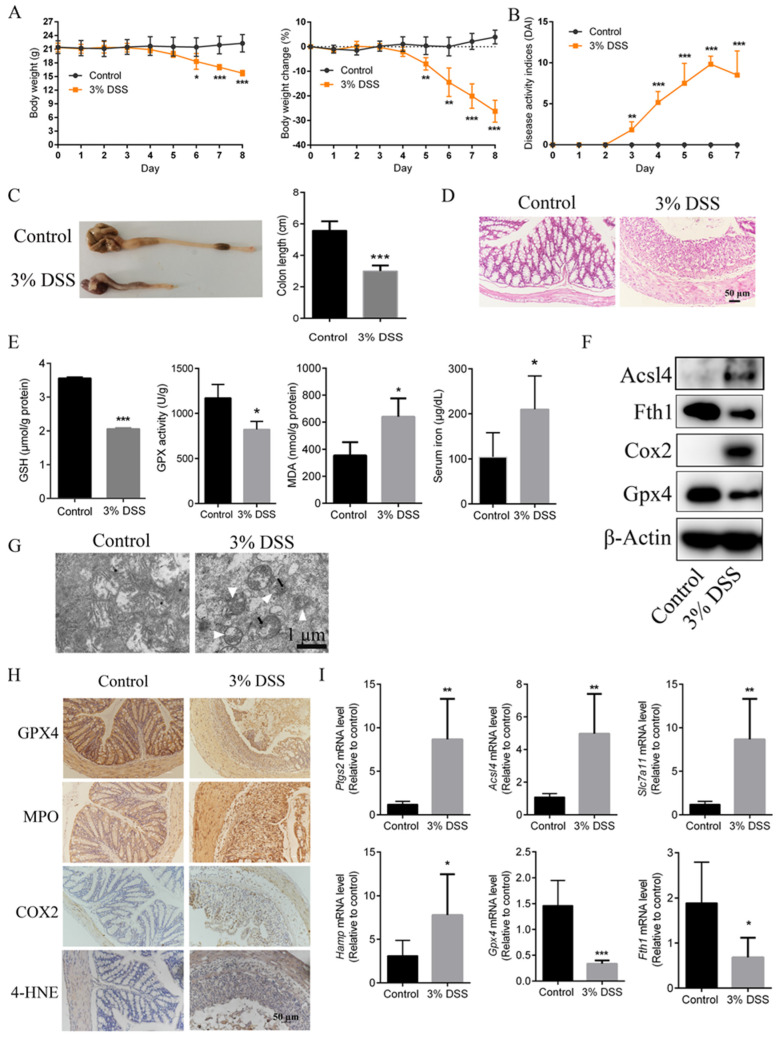
Ferroptosis is present in DSS-induced UC mice (*n* = 5). (**A**), Body weight and body weight changes in mice that received DSS treatment. (**B**), DAIs were determined according to scoring standards. (**C**), Colon length on the last day was measured and a histogram of colon length is shown. (**D**), H and E staining of colon tissues. Scale bar, 50 μm. (**E**), Levels of GSH, GPX activity and MDA in colonic tissues and serum iron levels of mice were examined using corresponding kits. (**F**), Protein expression levels of Acsl4, Fth1, Cox2 and Gpx4 were detected by western blotting. (**G**), Transmission electron microscopy of colon tissue mitochondria. Single white arrowheads indicate shrunken mitochondria; black arrowheads indicate increased mitochondrial membrane density. Scale bar, 1 μm. (**H**), Representative images of GPX4, MPO, COX2 and 4-HNE levels in colonic tissues of mice determined by immunohistochemistry staining. Scale bar, 50 μm. (**I**), Relative mRNA expression levels of *Ptgs2, Acsl4, Slc7a11, Hamp, GPX4* and *Fth1* were analyzed using RT-qPCR. Data are presented as the mean ± S.E.M. using Gapdh as a reference. * *p* < 0.05, ** *p* < 0.01, *** *p* < 0.001 versus mice from DSS group. 4-HNE, 4-hydroxynonenal; Acsl4, Acyl-CoA synthetase long-chain family member 4; Cox2, cyclooxygenase 2; DSS, dextran sulfate sodium; Fth1, ferritin heavy chain 1; GSH, glutathione; Gpx4, glutathione peroxidase 4; Hamp, hepcidin antimicrobial peptide; MDA, malondialdehyde; MPO, myeloperoxidase; Ptgs2, prostaglandin-endoperoxide synthase 2; Slc7a11, solute carrier family7 member 11.

Male C57BL/6J mice were treated with 3% DSS for seven days to induce an experimental UC mouse model. As shown in Figure 1A, mice challenged with 3% DSS exhibited a significant decrease in body weight from the fifth day compared with the control group mice. Furthermore, a marked increase in DAI from the third day (Figure 1C), markedly shortened colon length and the obvious histopathological changes in colon tissues after being challenged with DSS (Figure 1D) suggested the successful establishment of a UC animal model in the present study.

To verify the association between ferroptosis and the UC model used in the present study, some important ferroptosis markers were analyzed. Notably, DSS-induced UC in mice exhibited the typical hallmarks of ferroptosis. Decreased GSH content and GPX activity, and elevated MDA concentration and iron levels were found in DSS-induced UC mice (Figure 1E). Furthermore, the immunoblotting results demonstrated that DSS decreased the protein levels of Gpx4, ferritin heavy chain 1 (Fth1) and increased the protein levels of acyl-CoA synthetase long-chain family member 4 (Acsl4) and cyclooxygenase 2 (Cox2) (Figure 1F). In addition, the mitochondria in the colon tissues of mice treated with DSS were markedly smaller than those in the normal group (Figure 1G). High expression levels of COX2, MPO and 4-HNE and a low expression of GPX4 were observed in UC colon tissues examined by immunohistochemistry, reflecting that lipid peroxidation did occur in the colon tissues of UC mice (Figure 1H). Furthermore, significantly increased mRNA expression of prostaglandin-endoperoxide synthase 2 (*Ptgs2*), Acsl4, solute carrier family 7 member 11 (*Slc7a11*) and hepcidin antimicrobial peptide (*Hamp*) and notably decreased mRNA expression levels of *Gpx4* and *Fth1* were detected in the colon tissues of UC mice (Figure 1I). All aforementioned results demonstrated that ferroptosis occurs in the colonic tissues of UC mice.

### 2.2. Ferroptosis Occurred in Colon Macrophages of UC Mice

**Figure 2:**
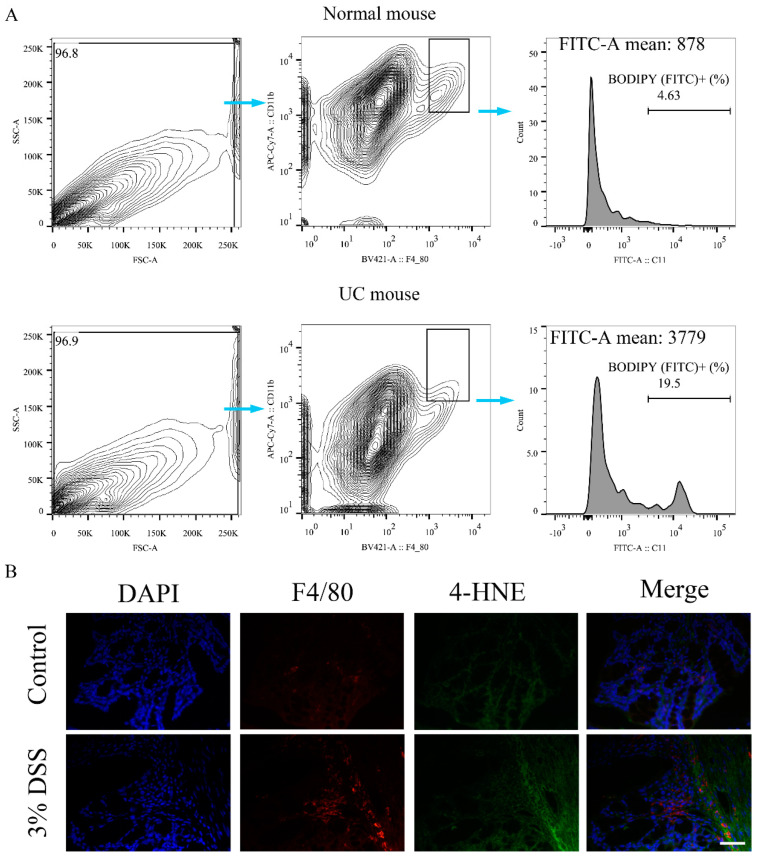
Ferroptosis occurs in colon macrophages. (**A**)**,** Representative flow cytometry images showing lipid peroxidation of colon macrophages of mice. (**B**), Representative images of F4/80 and 4-HNE levels in colonic tissues of mice were determined by colon tissue immunofluorescence staining, scale bar, 50 μm. 4-HNE, 4-hydroxynonenal; DAPI, 2−(4-Amidinophenyl)−6-indolecarbamidine dihydrochloride; DSS, dextran sulfate sodium.

To figure out whether ferroptosis occurs in colon macrophages, the lipid peroxidation of primary colon macrophages from control and UC mice was measured. The present results demonstrated that the colon macrophages of UC mice did undergo lipid peroxidation and the density was highly increased compared with the normal mice (Figure 2A). In addition, the colon tissue immunofluorescence results indicated that DSS increased the 4-HNE levels of colon macrophages in UC mice compared with the normal mice (Figure 2B). The results suggested that macrophages serve a major role in DSS-induced UC and macrophages maybe the main cells responsible for ferroptosis. Therefore, RAW264.7 cells and BMDMs were used in subsequent in vitro experiments.

### 2.3. Ferroptotic Cells Produced Pro-Inflammatory Cytokines and Triggered Inflammation

**Figure 3:**
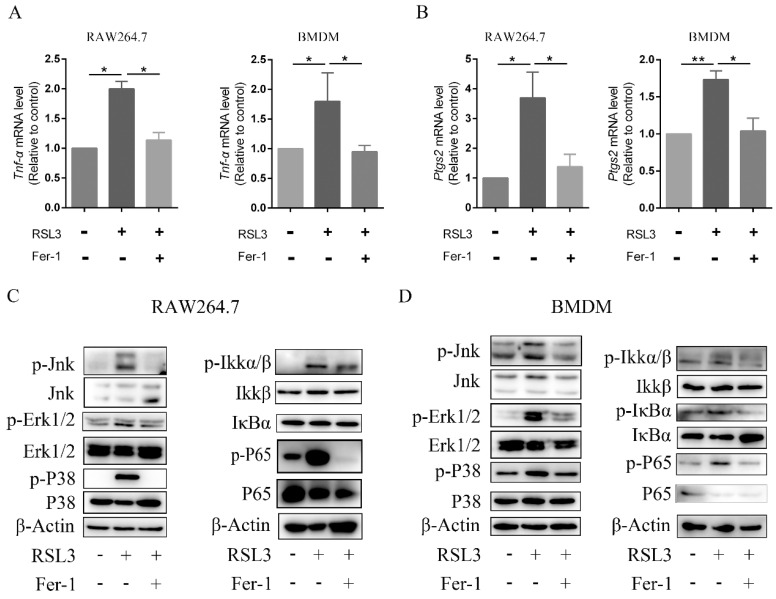
Inflammation is induced in ferroptotic macrophages. RAW264.7 macrophages and BMDMs were incubated in medium containing RSL3 (500 nM), or RSL3 (500 nM) combined with Fer-1 (400 nM) for 6 h. (**A**,**B**), mRNA expression levels of *Tnf-α* and *Ptgs2* in RAW264.7 macrophages and BMDMs were analyzed using RT-qPCR. Data are presented as the mean ± S.E.M. using Gapdh as a reference from three biologically independent samples. (**C**,**D**), Relevant protein levels of the MAPK signaling pathway and NF-κB pathways in RAW264.7 macrophages and BMDMs were analyzed by immunoblotting. β-Actin served as an internal reference. * *p* < 0.05, ** *p* < 0.01, versus RSL3 treatment group. BMDM, bone marrow-derived macrophage; Fer−1, ferrostatin−1; Ptgs2, prostaglandin-endoperoxide synthase 2; RSL3, ras-selective lethal small molecules 3; TNF-α, tumor necrosis factor α; Jnk, c-Jun N-terminal kinase; p-Jnk, phosphorylated-c-Jun N-terminal kinase; Erk1/2, extracellular regulated protein kinase1/2; p-Erk1/2, phosphorylated-extracellular regulated protein kinase1/2; Ikkα/β, inhibitor of kappa B kinase; p-Ikkα/β, phosphorylated-inhibitor of kappa B kinase; IκBα, inhibitor of nuclear factor kappa-B; p-IκBα, phosphorylated-inhibitor of nuclear factor kappa-B; P65, nuclear factor kappa-B; p-P65, phorylated-nuclear factor kappa-B.

To determine the relationship between inflammation and ferroptosis, RSL3, a ferroptosis activator, was used to induce ferroptosis of RAW264.7 macrophages and BMDMs. Notably, the mRNA levels of *Tnf-α* and *Ptgs2* were elevated in the RSL3 treatment group compared with the control group, while treatment with ferroptosis inhibitor Fer-1 decreased the raised expression levels of *Tnf-α* and *Ptgs2* in RAW264.7 cells (Figure 3A) and BMDMs (Figure 3B).

To further explore the molecular mechanisms, bioinformatics analysis was performed in the present study. A total of 10,868 inflammation-related genes, 282 ferroptosis-related genes and 4798 UC-related genes were identified using the GeneCards database. Furthermore, 121 common genes were obtained by cross-screening (Figure S2A) and subsequently GO and KEGG functional enrichment analyses were conducted. The results of the GO enrichment analysis revealed that the biological functions included ‘response to oxidative stress’, ‘inflammatory response’ and ‘lipid oxidation’ (Figure S2B), and the KEGG analysis revealed that signaling pathways such as ‘Ferroptosis’, ‘MAPK signaling pathway’ and ‘NF-kappa B signaling pathway’ serve important roles in inflammation, UC and ferroptosis, collectively (Figure S2C).

Based on the aforementioned analysis, the levels of proteins involved in the MAPK and NF-κB signaling pathways were detected using western blotting. Cells stimulated with RSL3 exhibited the robust phosphorylation of proteins involved in the MAPK and NF-κB signaling pathways, suggesting that the pathways were activated. Fer-1 treatment markedly reduced the levels of phosphorylated c-Jun N-terminal kinase (Jnk), phosphorylated extracellular regulated kinase 1/2 (Erk1/2), phosphorylated P38, phosphorylated IKKα/β, phosphorylated IκBα and phosphorylated P65 in RAW264.7 cells and BMDMs (Figure 3C,D). These data illustrated that ferroptotic cells might release pro-inflammatory cytokines and trigger an inflammatory response under certain conditions.

### 2.4. BCP Exerted an Inhibitory Effect on RSL3-Induced Macrophage Ferroptosis

Our screening experiment revealed that BCP had a good inhibitory effect on lipid peroxidation induced by RSL3 (Figure S3). To determine whether BCP acts as a promising ferroptosis inhibitor, experiments were carried out to explore the effect of BCP. Firstly, CCK8 assays were conducted to estimate the appropriate concentration of BCP in RAW264.7 cells and BMDMs. The results demonstrated that BCP was nontoxic (inhibition rate <5%) for RAW.2647 cells and BMDMs at concentrations ≤50 μM (Figure S4). Therefore, concentrations of 0–40 μM were selected as the test concentrations for further assays. 

**Figure 4:**
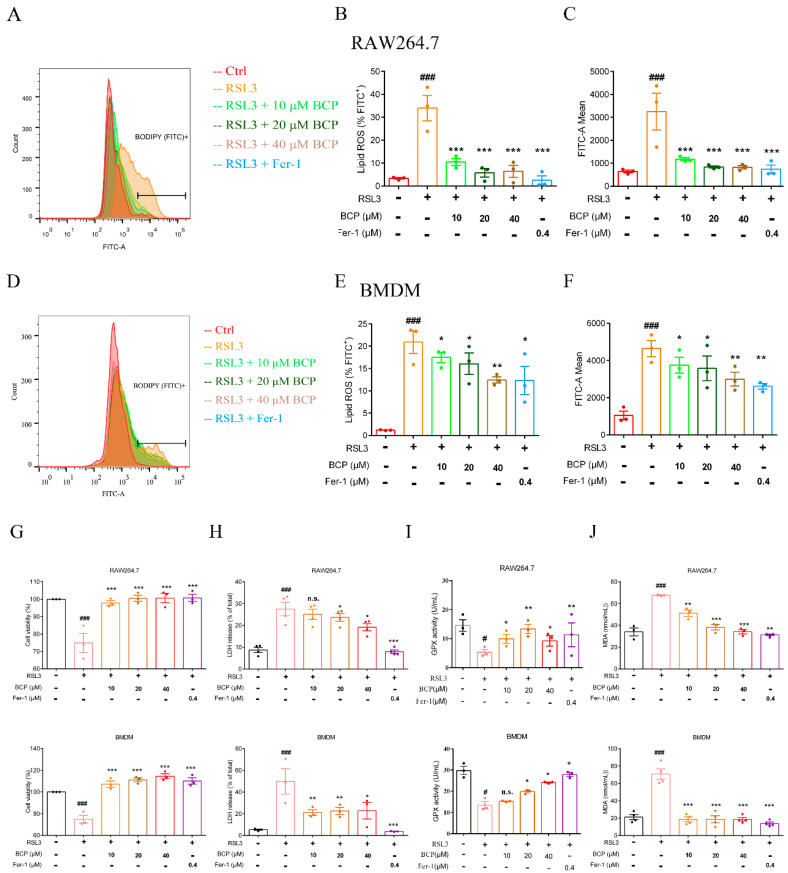
BCP exerts an inhibitory effect on RSL3-induced macrophage ferroptosis. RAW264.7 macrophages or BMDMs were treated with 500 nM RSL3 in the presence of different concentrations of BCP or 400 nM Fer-1 for 6 h. (**A**–**F**), Representative flow cytometry images, percentage of lipid ROS and fluorescence intensity showing lipid peroxidation of RAW264.7 macrophages and BMDMs after different treatments. (**G**), Cell viability was measured using a CCK−8 assay. (**H**), Release of LDH in cell supernatants was measured using LDH assay kit. (**I**), GPX activity was determined using GPX activity kit. (**J**), The concentrations of MDA were determined using TBARS assay kit. Data are presented as the mean ± S.E.M. at least three biologically independent samples. n.s., not significant, # *p* < 0.05, ### *p* < 0.001 versus control group; * *p* < 0.05, ** *p* < 0.01, *** *p* < 0.001 versus RSL3 treatment group. BCP, β-Caryophyllene; BMDM, bone marrow-derived macrophage; Fer−1, ferrostatin−1; GPX, glutathione peroxidase; LDH, lactate dehydrogenase; MDA, malondialdehyde; RSL3, ras-selective lethal small molecules 3.

**Figure 5:**
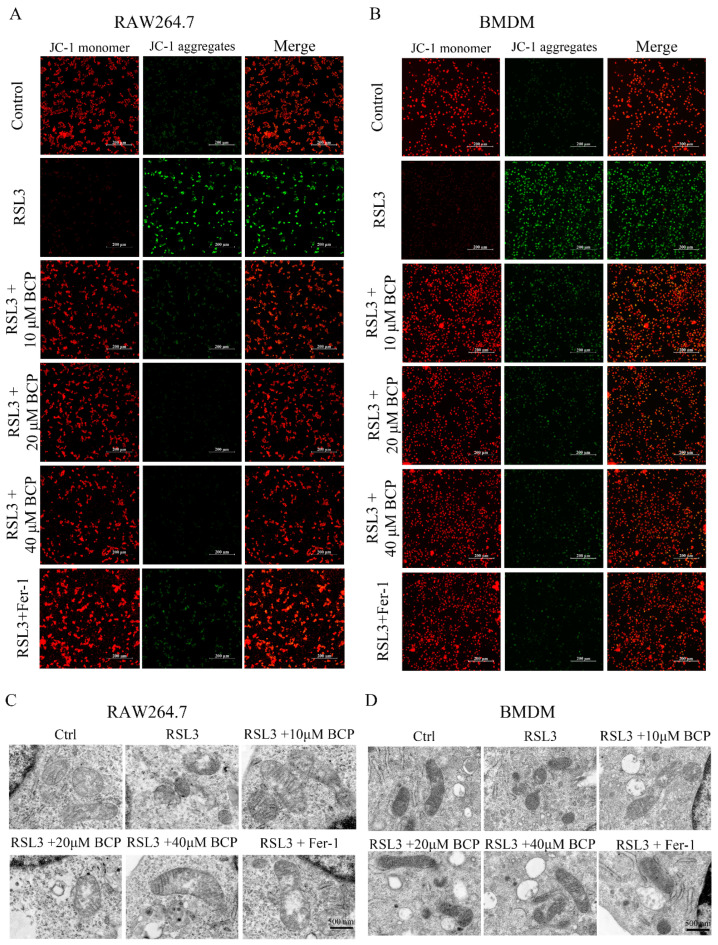
BCP ameliorates mitochondrial damage induced by RSL3. RAW264.7 macrophages or BMDMs were treated with 500 nM RSL3 in the presence of different concentrations of BCP or 400 nM Fer−1 for 6 h. (**A**,**B**), Representative JC-1 staining images showing mitochondrial membrane potential changes of RAW264.7 macrophages and BMDMs. Scale bar, 200 μm. (**C**,**D**), Transmission electron microscopy of RAW264.7 macrophage and BMDM mitochondria after different treatments. Scale bar, 500 nm. BCP, β-Caryophyllene; BMDM, bone marrow-derived macrophage; Fer−1, ferrostatin−1; RSL3, ras-selective lethal small molecules 3.

As an inhibitor of GPX4, RSL3 treatment notably induced lipid peroxidation in RAW264.7 cells and BMDMs based on C11-BODIPY staining, and this could be reversed by treatment with BCP at different concentrations and Fer-1 (Figure 4A–F and Figure S5A,B). In addition, BCP increased cell viability in RSL3-induced RAW264.7 cells and BMDMs (Figure 4G). When cell death occurs, the cell membrane is disrupted and LDH was released into the cell culture supernatant. Therefore, the contents of LDH in the supernatant can indicate the extent of ferroptotic death. As shown in Figure 4H, the release rate of LDH was also decreased after BCP at different concentrations and Fer-1 treatment. Furthermore, the decreased GPX activity and increased MDA production induced by RSL3 were markedly reversed by BCP at different concentrations and Fer-1 (Figure 4I,J). Additionally, BCP and Fer-1 treatment ameliorated the mitochondrial membrane potential decline induced by RSL3 (Figure 5A,B). Additionally, the ultrastructure of ferroptotic cells was observed using transmission electron microscopy. Notably, smaller mitochondria with increased membrane density were observed in RSL3-treated macrophages, while BCP and Fer-1 treatment ameliorated the mitochondrial morphological changes induced by RSL3 treatment (Figure 5C,D). In addition, BCP increased the mRNA levels of *Gpx4* and decreased *Hamp* mRNA expression levels in RAW264.7 cells and BMDMs (Figure S6A,B). Furthermore, RSL3 markedly decreased the protein levels of Gpx4 and Fth1 and observably augmented the protein levels of Cox2 and Alox5, whereas BCP reversed the protein levels. However, the levels of Acsl4 and Slc7a11 were not affected by RSL3 or BCP both in RAW264.7 cells and BMDMs (Figure S6C,D). These findings indicated that BCP exerted an inhibitory effect on RSL3-induced macrophage ferroptosis.

### 2.5. BCP Suppressed the Ferroptosis-Induced Inflammatory Response

**Figure 6:**
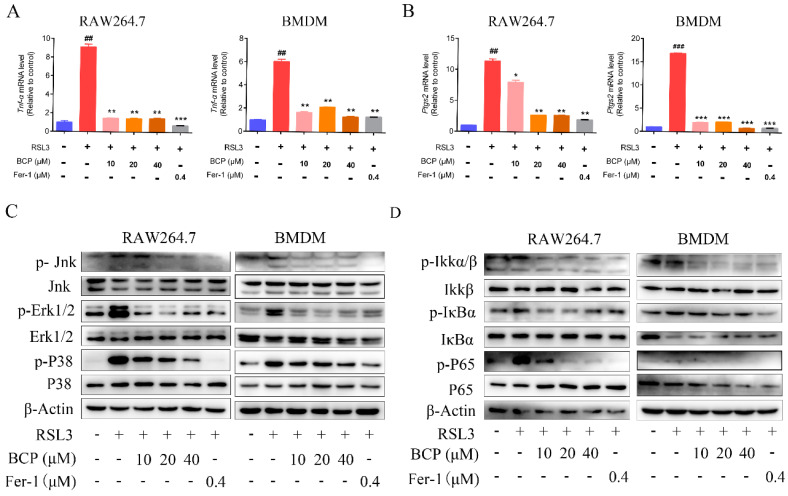
BCP treatment inhibits ferroptosis-induced inflammation. RAW264.7 macrophages or BMDMs were treated with 500 nM RSL3 in the presence of different concentrations of BCP or 400 nM Fer−1 for 6 h. (**A**,**B**), Relative mRNA levels of *Tnf-α* and *Ptgs2* in RAW264.7 macrophages and BMDMs were detected by RT-qPCR. Data are presented as the mean ± S.E.M. using Gapdh as a reference. ## *p* < 0.01, ### *p* < 0.001 versus control group; * *p* < 0.05, ** *p* < 0.01, *** *p* < 0.001 versus RSL3 treatment group. (**C**,**D**), Relevant protein levels of the MAPK and NF-κB signaling pathway in RAW264.7 macrophages and BMDMs were analyzed by immunoblotting. β-Actin was used as an internal reference. All data are representative of or combined from at least three independent experiments. BCP, β-caryophyllene; BMDM, bone marrow-derived macrophage; Fer−1, ferrostatin−1; Ptgs2, prostaglandin-endoperoxide synthase 2; RSL3, ras-selective lethal small molecules 3; TNF-α, tumor necrosis factor α; Jnk, c-Jun N-terminal kinase; p-Jnk, phosphorylated-c-Jun N-terminal kinase; Erk1/2, extracellular regulated protein kinase1/2; p-Erk1/2, phosphorylated-extracellular regulated protein kinase1/2; Ikkα/β, inhibitor of kappa B kinase; p-Ikkα/β, phosphorylated-inhibitor of kappa B kinase; IκBα, inhibitor of nuclear factor kappa-B; p-IκBα, phosphorylated-inhibitor of nuclear factor kappa-B; P65, nuclear factor kappa-B; p-P65, phorylated-nuclear factor kappa-B.

To further investigate the effect of BCP on the RSL3-induced inflammatory response, pro-inflammatory cytokines were examined. The results in Figure 6A,B indicated that the mRNA levels of *Tnf-α* and *Ptgs2* were increased in RSL3-treated RAW264.7 cells or BMDMs. Nevertheless, BCP or Fer-1 treatment effectively diminished the enhanced expression levels of these cytokines. 

Based on the results in Figure 3 and the bioinformatics analysis in Figure S2, immunoblotting analysis was performed to determine the effect of BCP on the MAPK and NF-κB signaling pathways activated by macrophage ferroptosis. Consistent with the aforementioned results, RSL3 stimulation increased the levels of phosphorylated Jnk, phosphorylated Erk1/2, phosphorylated P38, phosphorylated IKKα/β, phosphorylated IκBα and phosphorylated P65 in RAW264.7 cells and BMDMs. Conversely, BCP exerted an inhibitory effect on the ferroptosis-activated MAPK and NF-κB signaling pathways (Figure 6C,D). These results suggested that BCP suppressed the ferroptosis-induced inflammatory response. 

### 2.6. BCP Inhibited Macrophage Ferroptosis by Activating the CB2R

Previous studies have reported that BCP exerts multiple functions depending on its activation of CB2R. However, whether BCP inhibits ferroptosis via the activation of the CB2R is still unclear. Therefore, the CB2R antagonist AM630 was used to further explore the role of the CB2R in the protective ability of BCP against RSL3-induced ferroptosis. 

Schrodinger Maestro [31] was first used to perform molecular docking between BCP and the CB2R. Figure S7 shows that Van der Waals interactions exist between BCP and CB2R amino acid residues PHE87, ILE110, THR114, VAL113, ILE198, MET265 and SER285. BCP is connected to CB2R through a π-alkyl bond between the nine-membered ring and residue PHE183. Moreover, there are some π-alkyl interactions between BCP and CB2R residues PHE117, CYS288, TRP194, PHE281, VAL261, LEU262 and TRP258. The results suggested that BCP may bind to the CB2R. 

**Figure 7:**
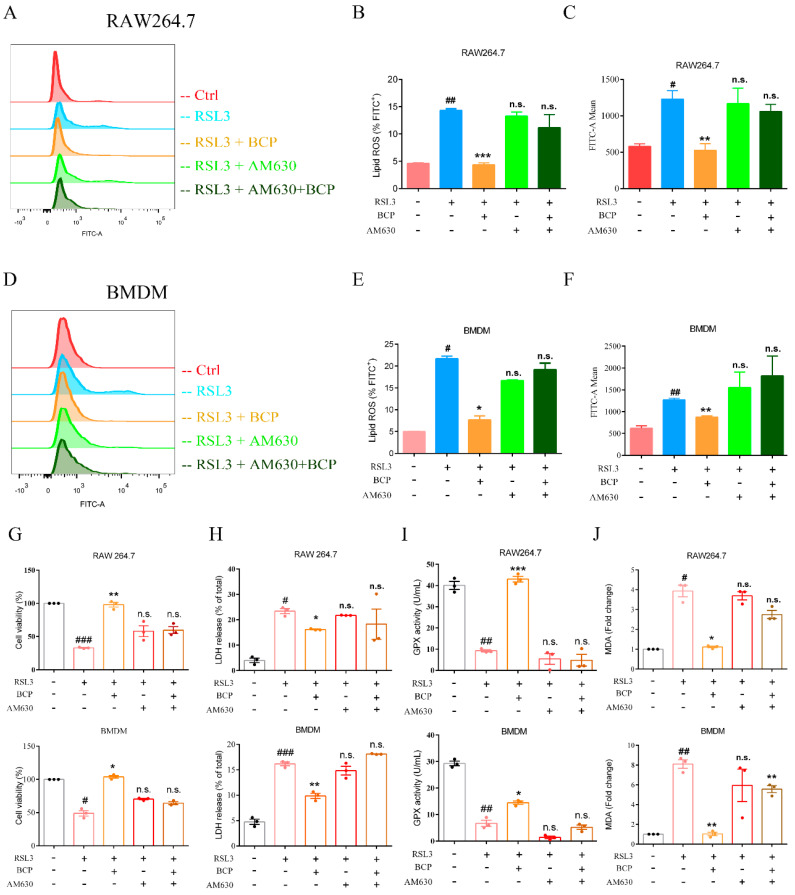
BCP inhibits macrophage ferroptosis by activating the CB2R. RAW264.7 macrophages or BMDMs were treated with 500 nM RSL3 in the presence of BCP (20 μM), CB2R antagonist AM630 (5 μM) or both AM630 and BCP for 6 h. (**A**–**F**), Representative flow cytometry images, percentage of lipid ROS and fluorescence intensity showing lipid peroxidation of RAW264.7 macrophages and BMDMs after different treatments. (**G**), Cell viability of RAW264.7 cells and BMDMs was measured using CCK-8 assay kit. (**H**), Release of LDH in cell supernatants was measured using LDH assay kit. (**I**), GPX activity was determined using GPX activity kit. (**J**), The concentrations of MDA were determined using TBARS assay. Data are presented as the mean ± S.E.M. n.s., not significant, # *p* < 0.05, ## *p* < 0.01, ### *p* < 0.001 versus control group; * *p* < 0.05, ** *p* < 0.01, *** *p* < 0.001 versus RSL3 treatment group. All data are combined from at least three independent experiments. AM630, 6-Iodopravadoline; BCP, β-caryophyllene; BMDM, bone marrow-derived macrophage; Fer−1, ferrostatin−1; GPX, glutathione peroxidase; LDH, lactate dehydrogenase; MDA, malondialdehyde; RSL3, ras-selective lethal small molecules 3.

**Figure 8:**
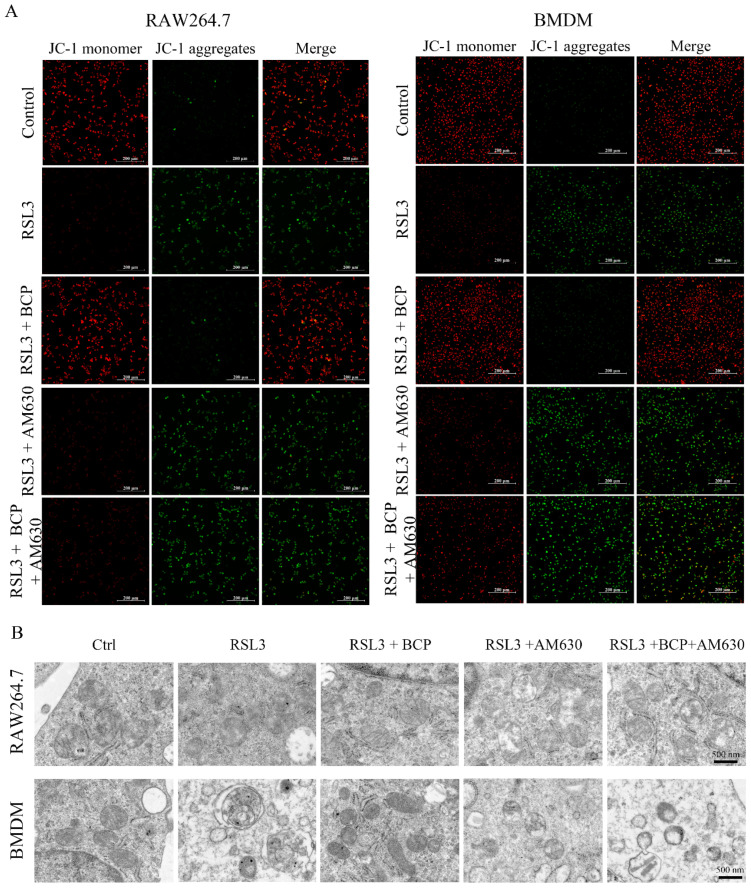
BCP ameliorates RSL3-induced mitochondrial damage via activation of the CB2R. RAW264.7 macrophages or BMDMs were treated with 500 nM RSL3 in the presence of BCP (20 μM), CB2R antagonist AM630 (5 μM) or both AM630 and BCP for 6 h. (**A**), Representative JC-1 staining images showing mitochondrial membrane potential changes of RAW264.7 macrophages and BMDMs. Scale bar, 200 μm. (**B**), Transmission electron microscopy of RAW264.7 macrophage and BMDM mitochondria after different treatments. Scale bar, 500 nm. AM630, 6-Iodopravadoline; BCP, β-caryophyllene; BMDM, bone marrow-derived macrophage; Fer-1, ferrostatin-1; RSL3, ras-selective lethal small molecules 3.

**Figure 9:**
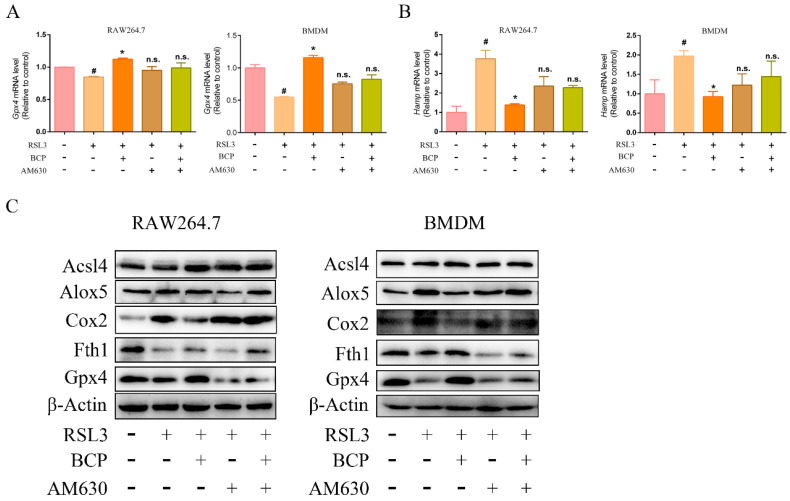
BCP regulates the expression of ferroptosis-related molecules by activating the CB2R. RAW264.7 macrophages or BMDMs were treated with 500 nM RSL3 in the presence of BCP (20 μM), CB2R antagonist AM630 (5 μM) or both AM630 and BCP for 6 h. (**A**), Relative mRNA levels of *Gpx4* and *Hamp* in RAW264.7 macrophages and BMDMs were detected by RT-qPCR. Data are presented as mean ± S.E.M. using Gapdh as a reference. n.s., not significant, # *p* < 0.05, control group; * *p* < 0.05, versus RSL3 treatment group. (**B**) Relevant protein levels of ferroptosis in RAW264.7 macrophages and BMDMs were analyzed by immunoblotting. β-Actin was used as an internal reference. (**C**) All data are representative of or combined from at least three independent experiments. Acsl4, Acyl-CoA synthetase long-chain family member 4; Alox5, lipoxygenase 5; AM630, 6−Iodopravadoline; BCP, β-caryophyllene; BMDM, bone marrow-derived macrophage; Cox2, cyclooxygenase 2; Fth1, ferritin heavy chain 1; Gpx4, glutathione peroxidase 4; Hamp, hepcidin antimicrobial peptide; RSL3, ras-selective lethal small molecules 3.

The lipid peroxidation induced by RSL3 was downregulated by BCP. However, treatment with AM630 alone did not alter RSL3-induced lipid peroxidation and AM630 reverses the BCP anti-lipid peroxidation effect (Figure 7A–F and Figure S8). Additionally, BCP treatment increased cell viability and GPX activity and decreased the LDH release and MDA content of RSL3-induced macrophages, which were abolished by AM630 (Figure 7G–J). Consistent with the aforementioned results, BCP treatment ameliorated mitochondrial membrane potential decline and the mitochondrial morphological changes induced by RSL3, which were reversed by AM630 (Figure 8). Moreover, BCP increased the mRNA levels of *Gpx4* and decreased *Hamp* mRNA expression levels in RAW264.7 cells and BMDMs, which were abolished by AM630 (Figure 9A). Furthermore, RSL3 markedly decreased the protein levels of Gpx4 and Fth1 and observably augmented the protein levels of Cox2 and Alox5, whereas BCP reversed the protein levels. However, AM630 notably abolished the inhibitory effect of BCP against macrophage ferroptosis (Figure 9B). These results suggested that BCP inhibited macrophage ferroptosis by activating the CB2R.

### 2.7. BCP Inhibited Ferroptosis-Induced Inflammation by Activating the CB2R

**Figure 10:**
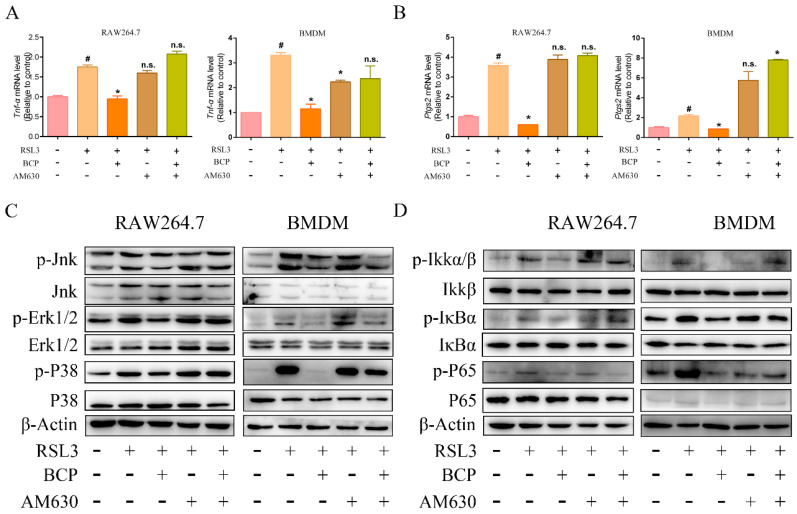
BCP inhibits ferroptosis-induced expression of inflammatory genes via activation of CB2R. RAW264.7 macrophages or BMDMs were treated with 500 nM RSL3 in the presence of BCP (20 μM), CB2R antagonist AM630 (5 μM) or both AM630 and BCP for 6 h. (**A**,**B**), Relative mRNA levels of *Tnf-α* and *Ptgs2* in RAW264.7 macrophages and BMDMs were detected by RT-qPCR. Data are presented as mean ± S.E.M. using Gapdh as a reference. n.s., not significant, # *p* < 0.05, control group; * *p* < 0.05, versus RSL3 treatment group. (**C**,**D**), Relevant protein levels of the MAPK signaling pathway and NF-κB pathways in RAW264.7 macrophages and BMDMs were analyzed by immunoblotting. β-Actin was used as an internal reference. All data are representative of or combined from at least three independent experiments. AM630, 6−Iodopravadoline; BCP, β-caryophyllene; BMDM, bone marrow-derived macrophage; Ptgs2, prostaglandin-endoperoxide synthase 2; RSL3, ras-selective lethal small molecules 3; TNF-α, tumor necrosis factor α; Jnk, c-Jun N-terminal kinase; p-Jnk, phosphorylated-c-Jun N-terminal kinase; Erk1/2, extracellular regulated protein kinase1/2; p-Erk1/2, phosphorylated-extracellular regulated protein kinase1/2; Ikkα/β, inhibitor of kappa B kinase; p-Ikkα/β, phosphorylated-inhibitor of kappa B kinase; IκBα, inhibitor of nuclear factor kappa-B; p-IκBα, phosphorylated-inhibitor of nuclear factor kappa-B; P65, nuclear factor kappa-B; p-P65, phorylated-nuclear factor kappa-B.

As shown in Figure 10A,B, BCP notably inhibited the mRNA expression levels of *Tnf-α* and *Ptgs2*, and treatment with AM630 alone did not alter the cytokine levels observed in the RSL3-treated macrophages; however, AM630 obviously abolished the anti-inflammatory effect of BCP. Additionally, BCP blocked the MAPK and NF-κB signaling pathways which were activated by ferroptosis; however, the effect was notably abolished by AM630 (Figure 10C,D). These results indicated that BCP inhibited ferroptosis-induced inflammation in a CB2R-dependent manner. 

### 2.8. BCP Effectively Mitigated DSS-Induced Ulcerative Colitis in C57BL/6J Mice via Activation of the CB2R

A schematic diagram of the BCP administration process in the animal experiments is shown in Figure S9A. The DAI score, an important indicator of UC, was calculated based on body weight change, stool consistency and bleeding. Therefore, symptomatic parameters including body weight, stool character and bleeding were recorded daily. As shown in Figure S9B–F, mice challenged with 3% DSS showed a significant decrease in body weight and a marked increase in DAI compared with control group mice. Following the administration of BCP, the body weight change and DAI score of DSS-induced mice were lower than those of the DSS group mice. However, the body weight change and DAI score of mice that were treated with AM630 alone or in combination with BCP did not show meaningful differences with the DSS group mice. Since another two typical symptoms of UC are a shortened colon, the colon length of every mouse was measured at the end of the study. The colon length of mice in the DSS group was observably shortened compared with those of control group mice. Notably, oral treatment with BCP, DSS-induced mice showed elevated colon length, which indicated the improved recovery of the colon. However, the protective effect of BCP against colon length reduction was abolished by AM630. In addition, the results of H and E staining demonstrated that the colon sections of normal mice had an intact epithelial cell surface with no histological abnormalities, while the colon sections of the DSS group mice exhibited defective epithelium, mucosal damage and crypt distortion with inflammatory cell infiltration. Following oral treatment with BCP, colon damage was alleviated in mice injured by DSS, but the effect was canceled out by AM630. (Figure S9G,H). These results demonstrated that BCP effectively mitigated DSS-induced UC in C57BL/6J mice by activating the CB2R. 

Based on the histological examination of colon tissues, UC colon tissues were infiltrated by numerous inflammatory cells, and thus, pro-inflammatory cytokines and chemokines were secreted by inflammatory cells to contribute to the UC progression. Therefore, the mRNA expression levels of pro-inflammatory cytokines, including *Tnf-α, Il-1β* and *Il-6* in colon tissues were detected. As expected, the colonic tissue mRNA levels of *Tnf-α, Il-1β* and *Il-6* were markedly elevated in the DSS group compared with the control group. Oral treatment with BCP markedly decreased the elevated mRNA expression levels of *Tnf-α, Il-1β* and *Il-6* and impaired the activation of MAPK and NF-κB signaling pathway; however, it was abolished by AM630 (Figure S10). These data clearly demonstrated that BCP effectively inhibited inflammatory responses in a functional CB2R-dependent way.

### 2.9. BCP Attenuated Ferroptosis Induced by DSS via Activation of the CB2R In Vivo

**Figure 11:**
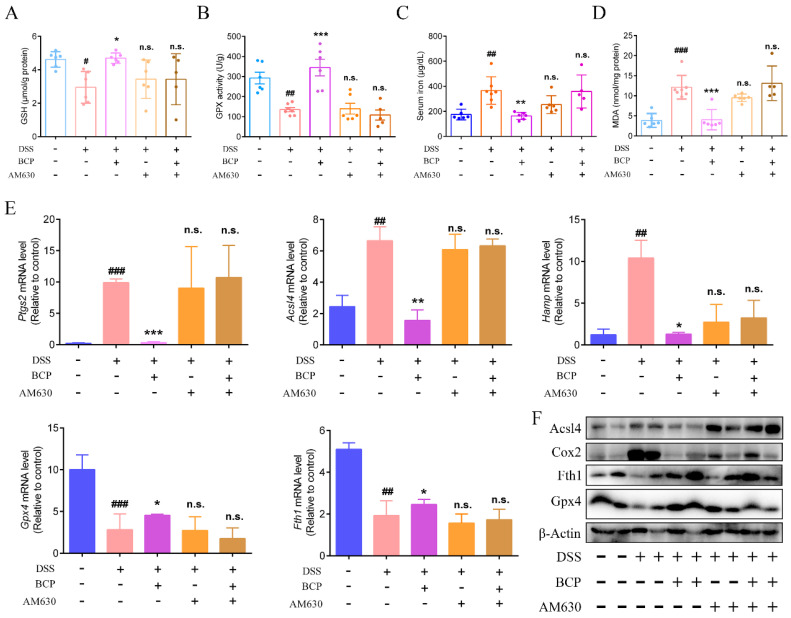
BCP attenuates ferroptosis induced by DSS by activating CB2R in vivo. Mice were given 3% DSS for 7 days and were treated with BCP (50 mg/kg, p.o.) alone, with the CB2R selective antagonist AM630 alone, or with AM630 (30 min before) plus BCP. (**A**–**D**), Content of GSH, GPX activity and MDA in colonic tissues and the levels of serum iron (*n* = 6) were examined using corresponding kits. (**E**), Relative mRNA levels of *Ptgs2, Acsl4, Hamp, Gpx4* and *Fth1* in colonic tissues of C57BL/6J mice were detected. Data are presented as the mean ± S.E.M. of 6 colonic tissue samples using Gapdh as a reference. (**F**), Acsl4, Cox2, Fth1 and Gpx4 protein expression in mice was determined by western blot analysis. β-Actin was used as an internal reference. n.s., not significant, # *p* < 0.05, ## *p* < 0.01, ### *p* < 0.001 compared with mice from control group; * *p* < 0.05, ** *p* < 0.01, *** *p* < 0.001 compared with mice from DSS group. Acsl4, Acyl −CoA synthetase long−chain family member 4; AM630, 6-Iodopravadoline; BCP, β-caryophyllene; Cox2, cyclooxygenase 2; DSS, dextran sulfate sodium; Fth1, ferritin heavy chain 1; GSH, glutathione; Gpx4, glutathione peroxidase 4; Hamp, hepcidin antimicrobial peptide; MDA, malondialdehyde; Ptgs2, prostaglandin-endoperoxide synthase 2.

The results in Figure 1 indicated that ferroptosis was present in the colonic tissues of UC mice. Consistent with the aforementioned results, the GSH content and GPX activity were lower, while the iron and MDA levels were higher in the UC mice compared with normal mice. After oral treatment with BCP, the GSH content and GPX activity were elevated and the iron and MDA levels were decreased. However, treatment with AM630 alone or in combination with BCP did not alter the ferroptosis parameters observed in the DSS-treated group (Figure 11A–D). In addition, the colonic mRNA expression levels of *Ptgs2*, *Acsl4* and *Hamp* were markedly upregulated in mice of the DSS group compared with the control group. Notably, DSS markedly suppressed colonic *Gpx4* and *Fth1* mRNA expression compared with the control group. Following treatment with BCP, the colonic mRNA expression levels of *Ptgs2, Acsl4, Hamp, Gpx4* and *Fth1* were reversed compared with those of mice in the DSS group. The western blot analysis of the key proteins of ferroptosis revealed that BCP markedly diminished the protein levels of Acsl4 and Cox2 induced by DSS and notably increased the protein levels of Fth1 and Gpx4 in colon tissue. However, AM630 signally abolished the anti-ferroptosis effect of BCP in vivo (Figure 11E,F). 

**Figure 12:**
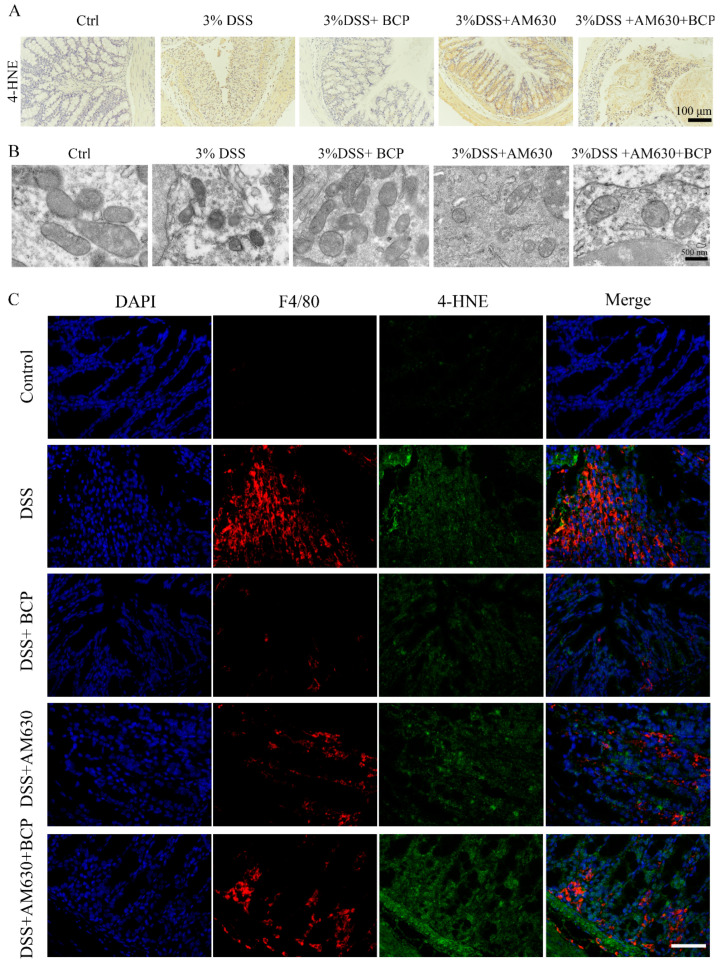
BCP ameliorates macrophage ferroptosis via activation of the CB2R in vivo (*n* = 6 in each group). (**A**), Representative images of immunohistochemical staining of colonic tissues for 4-HNE of colonic tissues. Scale bar, 100 μm. (**B**), Representative images of transmission electron microscopy of colonic tissues after different treatments. Scale bar, 500 nm. (**C**), Representative images of double immunofluorescence staining of colonic tissues for F4/80 and 4-HNE. Scale bar, 50 μm. 4-HNE, 4-hydroxynonenal; AM630, 6-Iodopravadoline; BCP, β-caryophyllene; DAPI, 2-(4-Amidinophenyl)-6-indolecarbamidine dihydrochloride; DSS, dextran sulfate sodium.

Additionally, high expression levels of 4-HNE in the UC colon tissues examined via immunohistochemistry were reversed by BCP. Furthermore, BCP ameliorated DSS-induced mitochondrial damage in the colon. In addition, the colon tissue immunofluorescence results revealed that DSS increased the 4-HNE levels of colon macrophages in UC mice compared with normal mice, while BCP treatment decreased the 4-HNE levels of colon macrophages. However, treatment with AM630 alone or in combination with BCP did not alter the ferroptosis parameters observed in the DSS-treated group (Figure 12). The results suggested that the pharmacological blockade of CB2R reverses the BCP anti-ferroptosis effect in vivo.

## 3. Discussion

UC is an inflammatory bowel disease with complex pathogenesis and is hard to cure. Compared with healthy individuals, patients who suffer from UC have an increased risk of colorectal cancer [32]. With the development of society and the increase in pressure, the number of patients with UC continues to increase, especially in Asia [33]. Although immunosuppressive agents are widely used in patients with IBD, a high percentage of patients do not benefit from these drugs [34,35]. Increasing evidence from several studies has revealed that cell death would be a critical therapeutic target for gastrointestinal diseases [19,36,37,38], which provides a novel perspective and potential for a pharmacological target in UC. Ferroptosis is a type of cell death that is independent of caspase and triggered by intracellular phospholipid peroxidation [8]. Previous studies by different research groups have reported that ferroptosis is involved in colitis [19,37,38,39]. The present results showed that decreased GSH content and GPX activity, and elevated MDA concentration and iron levels were found in DSS-induced UC mice. Ferroptotic cells were characterized by the abnormal shrinkage of mitochondrial morphology, the reduction or disappearance of the mitochondrial crest, and the increase in mitochondrial membrane density and membrane rupture [6,8]. Notably, the mitochondria in the colon tissues of mice treated with DSS were markedly smaller than those in the normal group. Moreover, shrunken mitochondria and increased mitochondrial membrane density were observed in the colon tissues of mice treated with DSS. These data indicated that ferroptosis participates in the pathological process of UC. 

In patients with colitis, the intestinal mucosal barrier is damaged, resulting in the increased uptake of luminal antigens, which contributes to active macrophages and other innate immune cells [1]. Active macrophages release substantial inflammatory chemokines to perpetuate the inflammatory cascade and further elicit cell death [40,41]. Therefore, we explored the role of ferroptosis in the colon macrophages of UC mice and found lipid peroxidation in the colon macrophages, which indicated that ferroptosis occurs in colon macrophages. Mounting evidence shows that ferroptotic cells induce pro-inflammatory cytokine production [9,42,43]. Through functional enrichment analysis and experimental verification, the present study demonstrated that MAPKs and NF-κB signaling pathways were activated in ferroptotic macrophages. Therefore, ferroptotic cells trigger an inflammatory response that further worsens ulcerative colitis. BCP exists in numerous plant essential oils and receives increasing attention from researchers since it is an edible spice and is a fully selective agonist of CB2R [21,44,45]. Although many studies have reported various functions of BCP, the effects of BCP on macrophage ferroptosis remain to be elucidated. In the present study, we found that BCP decreased pro-ferroptotic factors and increased anti-ferroptotic factors in RSL3-reduced macrophages, indicating that BCP may have an inhibitory effect on macrophage ferroptosis. Additionally, ferroptotic macrophages increase the mRNA expression of *Tnf-α* and *Ptgs2* and activate MAPK and NF-κB signaling cascades. Nevertheless, BCP treatment markedly inhibited the inflammatory response caused by ferroptotic macrophage. In addition, through oral treatment with BCP, the GSH content and GPX activity were elevated, and iron and MDA levels were decreased in UC mice. Moreover, the IHC, IF and TEM results also revealed the anti-ferroptosis effects of BCP in vivo. Further research found that the CB2R antagonist AM630 markedly abolished the macrophage ferroptosis inhibitory effect of BCP both in vitro and in vivo, which indicated that BCP inhibited ferroptosis via the activation of the CB2R. 

In conclusion, the present study provided evidence that macrophage ferroptosis is closely related to UC, and BCP acts as a ferroptosis inhibitor that suppresses lipid peroxidation and inflammation, ameliorating UC from ferroptosis. The evidence suggests that targeting macrophage ferroptosis might be a promising approach to delay the progression of UC and that BCP could be a promising candidate, providing a pharmacological rationale for its pharmacotherapeutic application and pharmaceutical development as a drug for therapeutic and preventive applications in UC treatment. Thus, the study linked cell death-immune crosstalk to UC inflammation, not only illuminating the mechanism, but also providing targets for UC treatment. In addition, the study demonstrated a novel mechanism underlying the protective effect of BCP treatment against UC for the first time. 

## 4. Materials and Methods

### 4.1. Chemicals and Reagents

BCP (>95% purity) was purchased from Stanford Analytical Chemicals Inc. (Stanford, CA, USA). Moreover, (1S/3R) RSL3 (RSL3) was purchased from MedChem Express (Monmouth Junction, NJ, USA). Ferrostatin-1 (Fer-1) was purchased from Topscience Co., Ltd. (Shanghai, China). DSS (molecular weight, 36,000–50,000 kDa) was obtained from MP Biomedicals LLC (Irvine, CA, USA). Pharmaceutical-grade corn oil was purchased from Shanghai Aladdin Biochemical Technology Co., Ltd. (Shanghai, China). BCP and Fer-1 were solubilized in dimethyl sulfoxide (DMSO), and diluted with phosphate-buffered saline (PBS) as needed. The final DMSO concentration was 0.1% (*v*/*v*). The major small-molecule compounds in the essential oil of Artemisia argyi used in this study included β-Caryophyllene (20 μM, CYH-207225, Stanford Analytical Chemicals Inc.), 4-Terpinenol (20 μM, 111967, National Institutes for Food and Drug Control (NIFDC, Beijing, China)), Eupatilin (20 μM, S3846, Selleck), Cineol (20 μM, 110788, NIFDC), α-Terpineol (20 μM, 111859, NIFDC), Borneol (20 μM, 111688, NIFDC), Camphor (20 μM, 111749, NIFDC), L-Carveol (20 μM, AF180610, Stanford Analytical Chemicals Inc.) and α-Thujone (20 μM, SM170318, Stanford Analytical Chemicals Inc.). The molecular structures of these compounds are shown in Figure S1. Information regarding the primary antibodies used is provided in Table S1. All other ordinary reagents were obtained from Guangzhou Chemical Reagent Factory (Guangzhou, China).

### 4.2. Cell Culture

RAW 264.7 cells were obtained from the Cell Bank of the Chinese Academy of Sciences (Shanghai, China). Cells were cultured in Dulbecco’s modified Eagle’s medium (DMEM) with 10% Fetal bovine serum (FBS, #10100-147; Gibco, Grand Island, NY, USA) and 1% penicillinstreptomycin (#15070063; Gibco) in a humidified atmosphere at 37 °C with 5% CO_2_. Bone marrow-derived macrophages (BMDMs) were isolated as described in a previous study [46] with some differences. Briefly, the tibia and femur of 8–12-week old male C57BL/6 mice were collected and then the bone marrow was flushed out using a 1 mL syringe with α-MEM (#12571-500, Gibco). The bone marrow cells were collected in a 15 mL tube and centrifuged at 300× *g* for 5 min. Cells were resuspended in fresh α-MEM supplemented with 10% FBS, 2 mM L-Glutamine (#25-005-Cl; Corning, NY, USA), and 1% streptomycin plus penicillin, plated in 60 mm Petri dishes and cultured at 37 °C with 5% CO_2_ overnight. The next day, nonadherent cells were collected, the density was adjusted to 3 × 10^6^ cells/mL using α-MEM growth medium containing macrophage colony-stimulating °factor (#315-02; Peprotech, La Jolla, CA, USA) and cells were seeded in a cell culture dish in a humidified atmosphere with 5% CO_2_ at 37 °C. RAW264.7 macrophages or BMDMs were treated with 500 nM RSL3 in the presence of different concentrations of BCP or 400 nM Fer-1 for 6 h and then used for further experiments.

The isolation of colon tissue macrophages was based on the reference [47] with slight modification. In brief, rinsed colon tissues were cut into 1-mm slices, then put into a sterile centrifugal tube and shaken at 37 °C for 30 min in D-Hanks’ buffer with 3 mM ethylenediaminetetraacetic acid. Subsequently, the colon slices were washed twice with Hanks’ Balanced Salt Solution and then shaken at 37 °C in Roswell Park Memorial Institute (RPMI) 1640 medium containing 1% streptomycin penicillin, 10% FBS, 200 U/mL collagenase (#C5138; Sigma-Aldrich, St. Louis, MI, USA) and 5 U/mL deoxyribonuclease (#LS002138; Worthington, Columbus, OH, USA). After 2 h, single-cell suspensions were harvested after sterile 70 μm primary cell filter filtration. Cells were counted and further sorted according to cell-surface markers of macrophages, such as F4/80^+^ and CD11b^+^ by using flow cytometry.

### 4.3. Cell Viability Assay

RAW264.7 cells or BMDMs were cultured overnight in a 96-well plate containing 100 µL growth medium (DMEM or α-MEM with 10% FBS) and exposed to a range of BCP concentrations for 24 h. Subsequently, 10 µL Cell Counting Kit-8 (CCK-8) solution (#CK04; Dojindo, Kumamoto, Japan) was added to each well and the plate was incubated for 1 h in the dark at 37 °C. The plates were gently shaken for 15 min at room temperature, before the optical density (OD) was recorded at 450 nm using an enzyme immunoassay reader (Bio-Rad Laboratories, Inc., Hercules, CA, USA). The cell viability at each BCP concentration was normalized to that of BCP-free control cells.

### 4.4. Animal Model of UC and Pharmacological Treatments

Specific pathogen-free male C57BL/6 mice (7–9 weeks old; 19–21 g) were purchased from Beijing Vital River Laboratory Animal Technology Co., Ltd. (Beijing, China). All mice were housed in a specific pathogen-free and temperature-controlled room with a 12 h/12 h light/dark cycle and had free access to food and water. 

After 10 days of acclimation, the mice were randomly assigned to five groups (*n* = 6 each): control, model (3% DSS), BCP (50 mg/kg body weight), AM630 (10 mg/kg body weight) [24] and AM630 + BCP. All mice (except those in the control group) were provided with a solution of filtered water containing 3% (*w*/*v*) DSS ad libitum during the experiment period. The mice in the control group received only normal drinking water. For the treatment groups, BCP was dissolved in corn oil at doses of 50 mg/kg and administered orally once a day. The mice in the AM630 group were injected intraperitoneally with AM630 (10 mg/kg). The mice in the AM630 + BCP group received an intraperitoneal injection of AM630 (10 mg/kg) given 30 min before the BCP (50 mg/kg). During the study, symptomatic parameters including body weight, stool character and bleeding were recorded daily. The disease activity index (DAI) was calculated according to our previous study [48]. After 8 days of treatment, all mice were anesthetized with pentobarbital sodium. The blood and colon samples were collected for subsequent analysis. The collected blood was left to coagulate for 20 min at room temperature and centrifuged for 15 min at 3000 g to obtain the serum. All experiments were carried out in accordance with the National Institutes of Health’s Guide for the Care and Use of Laboratory Animals. All animal protocols were approved by the Institutional Animal Care and Use Committee (IACUC) of Jinan University (Approval number: 20201008-02).

### 4.5. Assessment of GSH and GPX Activity, and MDA and Iron Levels

GSH and GPX activity, and MDA and iron levels were measured according to the manufacturer’s protocol using the GSH/GSSG Ratio Detection Assay Kit (#ab138881; Abcam, Cambridge, UK), GPX Activity Assay Kit (#AKPR014M; Beijing Boxbio Science and Technology Co., Ltd., Beijing, China), TBARS assay kit (#10009055; Cayman Chemical Company, Ann Arbor, MI, USA,) and Iron Assay Kit (#ab83366; Abcam, Cambridge, UK), respectively. The total protein concentration was measured using the Enhanced BCA Protein Assay Kit (#P0009; Beyotime Institute of Biotechnology, Haimen, China).

### 4.6. Lactate Dehydrogenase (LDH) Release Assay

The release levels of LDH in the culture medium were measured using the LDH cytotoxicity detection kit (#C0017; Beyotime Institute of Biotechnology, Haimen, China) according to the manufacturer’s instructions [49]. In brief, one group of wells was selected as a “maximal LDH release rate group.” Before treating cells as per the method in the “Cell culture and stimulation” section, cells were washed with PBS and the medium was replaced with a serum-free medium. LDH release reagent was added to the wells in the ‘maximal LDH release rate group’ 1 h before the end of treatment. At the end of treatment, the plate was centrifuged at 400× *g* for 5 min at room temperature. A total of 120 μL supernatant was transferred to a new plate. Subsequently, a 2-p-iodophenyl-3-nitrophenyl tetrazolium chloride and lactic acid solution was added to each well and the plate was incubated in the dark at room temperature for 30 min. The OD at 490 nm was measured and the release rate of LDH was calculated according to the following equation:

Release rate of LDH = (OD of treated sample-OD of control)/(OD of ‘maximal LDH release rate group’-OD of control) × 100%

### 4.7. Gene Expression Analysis Using Reverse Transcription-Quantitative PCR (RT-qPCR)

Total RNA from colon tissues or cultured cells, which were treated as indicated, was isolated using TRIzol Reagent (#DP424; Tiangen Biotech Co., Ltd., Beijing, China) and RNA concentrations were measured using a NanoPhotometer™ P-Class P330 (Implen GmbH, Munich, Germany). The extracted RNA (1000 ng) was reverse-transcribed to cNDA (10 μL) using an Evo M-MLV RT Premix kit (#AG11706; ACCURATE Bio, Inc., Hunan, China). RT-qPCR assays were performed in a CFX96 Touch Real-Time PCR Detection System (Bio-Rad Laboratories, Inc., Hercules, CA, USA) and the SYBR^®^ Green Pro Taq HS qPCR Kit (#AG11702; ACCURATE Bio, Inc., Hunan, China) according to the manufacturer’s instructions. The relative gene expression levels were normalized to the internal housekeeping gene, Gapdh. All data are from at least three independent experiments. The gene-specific primers are listed in Table S2. 

### 4.8. Immunoblotting

The western blot analysis used in the present study was similar to the procedure in our previous study [50] with some modifications. Briefly, the colon tissues or cultured cells with the indicated treatment were harvested and lysed in RIPA lysis Buffer (#P0013B; Beyotime Institute of Biotechnology, Haimen, China) containing a protease inhibitor (phenylmethylsulfonyl fluoride; #ST506; Beyotime Institute of Biotechnology, Haimen, China) and phosphatase inhibitor cocktail (#P1082; Beyotime Institute of Biotechnology, Haimen, China). The lysates were normalized to equal amounts of protein using the Enhanced BCA Protein Assay Kit (#P0009; Beyotime Institute of Biotechnology, Haimen, China). The proteins were separated by sodium dodecyl sulfate polyacrylamide gel electrophoresis (8–12% acrylamide) and then transferred to 0.22 μm polyvinylidene difluoride membranes (#ISEQ00010; MilliporeSigma, Burlington, MA, USA), followed by blocking with 5% skimmed milk in Tris-buffered saline-Tween 20 [150 mM NaCl, 50 mM Tris-HCl, pH 7.6, and 0.1% (*v*/*v*) Tween-20] for 1 h. The membranes were incubated with primary antibodies overnight at 4 °C, followed by incubation with anti-mouse (1:5000 dilution) or anti-rabbit (1:8000 dilution) horseradish peroxidase (HRP)-conjugated secondary antibodies for 1 h at room temperature. The blots were visualized using enhanced chemiluminescence reagent (#WBKLS0500; MilliporeSigma, Burlington, MA, USA) and imaged with a Tanon 5200 image analysis system (Tanon Science and Technology Co., Ltd., Shanghai, China).

### 4.9. Lipid Peroxidation Assay Using Flow Cytometry

The cellular lipid peroxidation was measured using flow cytometry (BD FACSCanto) with C11-BODIPY (581/591) (#D3861; Invitrogen, Thermo Fisher Scientific, Inc., Waltham, MA, USA) staining. The RAW264.7 cells or BMDMs were treated with RSL3, with or without BCP or Fer-1 for 6 h. Before the end of the treatment time, the culture medium was replaced with 1 mL fresh medium containing 10 μM C11-BODIPY (581/591) dye for 30 min in a dark place. Subsequently, the cells were washed twice with PBS, digested with trypsin and resuspended with PBS. The cell suspension was filtered through a 70 μm cell strainer and analyzed using a flow cytometer employing an excitation wavelength of 488 nm.

### 4.10. Histological Examination

The histological examination performed in the present study was similar to the procedure in our previous study [50]. Briefly, fresh colon tissue (1.5 × 1.5 × 0.5 cm) was fixed in 4% paraformaldehyde for 8 h at room temperature, dehydrated using an alcohol gradient and embedded in paraffin. The tissue was then sectioned into 3 μm slices and stained with hematoxylin and eosin (H and E) and assessed for inflammation using a light microscope (Nikon Corporation, Tokyo, Japan). The histology scores represent the sum of each histological alteration outlined below. This system assesses inflammation, epithelial defects, crypt atrophy and dysplasia by giving each parameter a separate score for severity [48].

### 4.11. Immunohistochemistry

Immunohistochemistry was performed on paraffin-embedded sections. The sections of the mice colon tissue were deparaffinized, rehydrated and antigen-retrieved for 15 min in sodium citrate at room temperature. Subsequently, the endogenous peroxidase activity was blocked by using 3% hydrogen peroxide solution and the sections were blocked with 3% bovine serum albumin (BSA). Afterward, the colon sections were incubated with primary antibodies, including COX2 (1:500), GPX4 (1:400), 4-HNE (1:400), or myeloperoxidase (MPO; (1:400) overnight at 4 °C and then labeled with HRP-conjugated antibody at room temperature for 1 h. PBS was used as negative control instead of primary antibody to ensure the specific binding of antibodies to the target protein. Stained sections were analyzed and captured under a light microscope (Nikon Corporation, Tokyo, Japan). 

### 4.12. Immunofluorescence

Paraffin sections of the mouse colon tissue were deparaffinized using dimethylbenzene and ethanol and rehydrated. Subsequently, the colon tissue sections were placed in a box filled with EDTA antigen repair buffer (PH8.0) to repair antigen, and blocked with 3% BSA for 1 h. Afterward, the colon tissue sections were incubated with the primary antibodies, including F4/80 and 4-HNE overnight at 4 °C, followed by incubation with the conjugated secondary antibody at room temperature in the dark for 1 h. PBS was used as a negative control instead of a primary antibody to ensure the specific binding of antibodies to the target protein. The nuclei were counterstained with DAPI in the dark for 10 min at room temperature.

The immunofluorescence staining of the cells was performed as described subsequently. RAW264.7 cells or BMDMs were treated with RSL3, with or without BCP, AM630 or Fer-1. Before the end of the treatment time, the culture medium was replaced with 1 mL fresh medium containing 10 μM C11-BODIPY (581/591) dye or JC-1 fluorescent probe for 30 min in the dark at 37 °C. Stained sections were analyzed and images were captured under a light microscope (Nikon Corporation, Tokyo, Japan). 

### 4.13. Transmission Electron Microscopy

Transmission electron microscopy was performed using standard procedures by Wuhan Servicebio Biotechnology [15]. In brief, cells were fixed with 2.5% electron microscope fixing solution for 4 h. Subsequently, cells were embedded with resin, dehydrated, and cut into ultrathin sections (60–80 nm) using an ultramicrotome (Leica UC7; Leica). Sections were stained with uranyl acetate in pure ethanol for 15 min and then stained with lead citrate for 15 min. Images were acquired using a Transmission Electron Microscope (HT7700; Hitachi, Ltd., Tokyo, Japan). 

### 4.14. Statistical Analysis

Results were analyzed using GraphPad Prism 6.02 (GraphPad Software, Inc., La Jolla, CA, USA). All data are presented as the mean ± SEM and are representative of at least three different experiments or biological replications. Statistical significance was determined using Student’s *t*-test for two groups and one-way ANOVA Tukey’s multiple comparisons test for multiple groups. A significant difference was indicated by *p* ≤ 0.05, 0.01, or 0.001.

## Data Availability

Not applicable.

## Supplementary Materials

The following supporting information can be downloaded at: https://www.mdpi.com/article/10.3390/ijms232416055/s1.
