# Supplementary material for: β-Caryophyllene Acts as a Ferroptosis Inhibitor to Ameliorate Experimental Colitis
Source: Int J Mol Sci. 2022 Dec 16;23(24):16055. doi: 10.3390/ijms232416055 (PMC9784863; doi:10.3390/ijms232416055)
Supplement: Supplementary file 1 [file ijms-23-16055-s001.zip › Supplementary Tables and Figures-ijms.pdf]

β-Caryophyllene acts as a ferroptosis inhibitor to ameliorate experimental colitis

Supplementary Tables

Table S1 List of primary antibodies for immunoblot, immunohistochemistry or Immunofluorescence

Name	Supplier	item number
4-HNE	Abcam	Cat # ab46545
ACSL4	Santa Cruz	Cat # sc-271800
APC anti-mouse F4/80	Biolegend	Cat # 123115
APC/Fire™ 750 anti-mouse/human CD11b	Biolegend	Cat # 101262
Brilliant Violet 421 anti-mouse F4/80	Biolegend	Cat # 123131
COX2	CST	Cat # 12282
Erk1/2 MAPK (137F5)	CST	Cat # 4695
F4/80	Servicebio	Cat # GB113373
FTH1 (D1D4)	CST	Cat # 4393
GPX4	Abcam	Cat # ab125066
IKKβ (D30C6)	CST	Cat # 8943
IκBα (L35A5)	CST	Cat # 4814
JNK	CST	Cat # 9252
NF-κB p65 (D14E12)	CST	Cat # 8242
p38 MAPK	CST	Cat # 9212
Phospho-Erk1/2 MAPK (Thr202/Tyr204)	CST	Cat # 4370
Phospho-IKKα (Ser176)/IKKβ (Ser177)	CST	Cat # 2078
Phospho-IκBα (Ser32/36)	CST	Cat # 9246
Phospho-SAPK/JNK (Thr183/Tyr185)	CST	Cat # 9255
Phospho-NF-κB p65 (Ser536)	CST	Cat # 3033
Phospho-p38 MAPK (Thr180/Tyr182)	CST	Cat # 4511
xCT/SLC7A11 (D2M7A)	CST	Cat # 12691
β-Actin	CST	Cat # 4970

Table S2. The RT-qPCR sequences used in the study

Genes	Primer sequences (5′→3′)
Acsl4	F: CTCACCATTATATTGCTGCCTGT<br>R: TCTCTTTGCCATAGCGTTTTTCT
Fth1	F: TGCCTCCTACGTCTATCTGTC<br>R: GTCATCACGGTCTGGTTTCTTT
Gpx4	F: GCCTGGATAAGTACAGGGGTT<br>R: CATGCAGATCGACTAGCTGAG
Gapdh	F: TGTGTCCGTCGTGGATCTGA<br>R: CCTGCTTCACCACCTTCTTGA
Hamp	F: TGCCTGTCTCCTGCTTCT<br>R: TGTCTGCCCTGCTTTCTT
Il-1β	F: CTGGTGTGTGACGTTCCCATTA<br>R: CCGACAGCACGAGGCTTT
Il-6	F: CCAGTTGCCTTCTTGGGACT<br>R: GGTCTGTTGGGAGTGGTATCC
Ptgs2	F: GGCCATGGAGTGGACTTAAA<br>R: ACCTCTCCACCAATGACCTG
Slc7a11	F: CTTTGTTGCCCTCTCCTGCTTC<br>R: CAGAGGAGTGTGCTTGTGGACA
Tnf-α	F: CAAAATTCGAGTGACAAGCCTG<br>R: GAGATCCATGCCGTTGGC

Supplementary figures

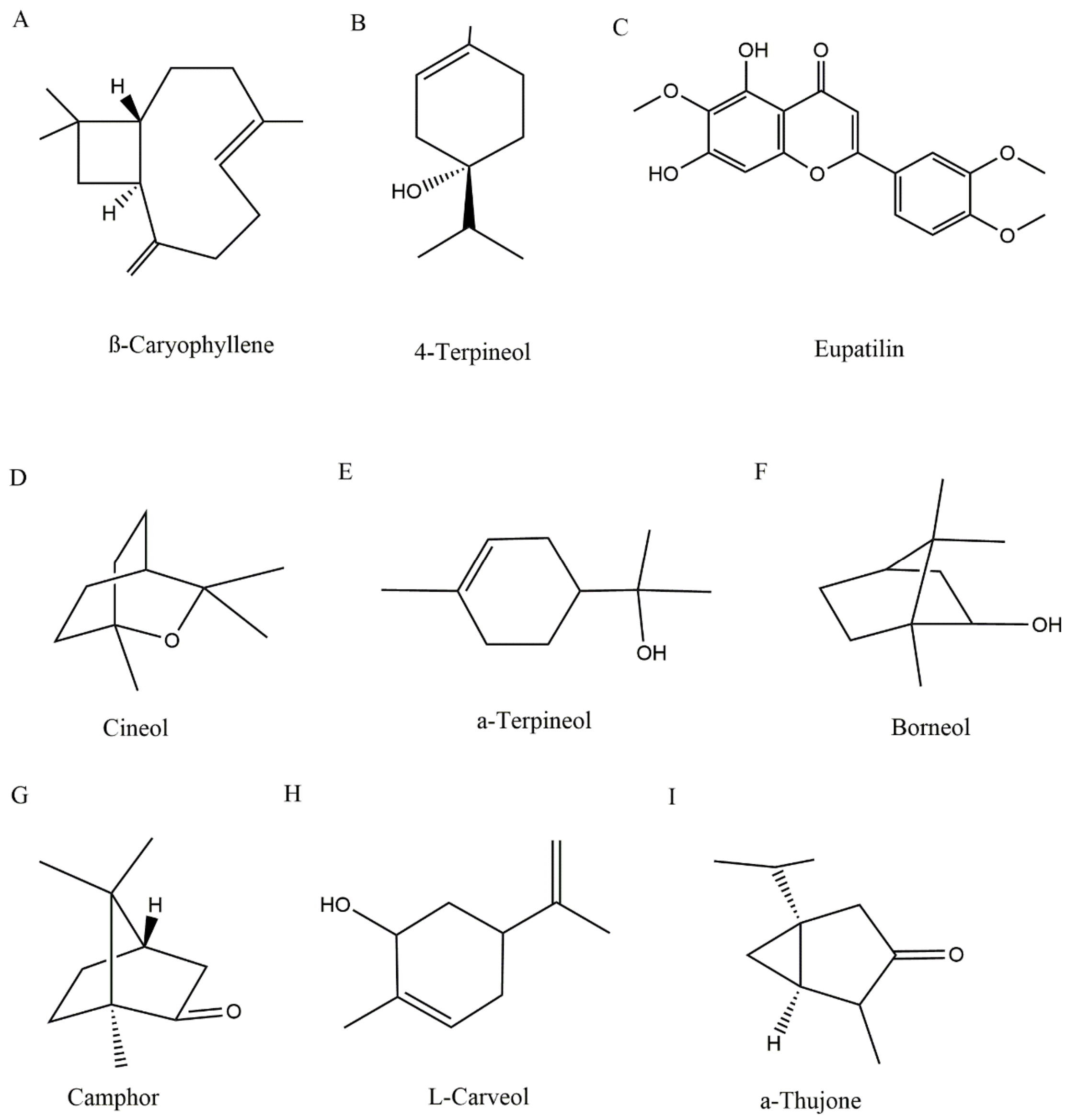

Figure S1. The major small molecule compounds in the essential oil of Artemisia argyi used in this study. Molecular structure of (A), β-Caryophyllene; (**B**), 4-Terpinenol; (**C**), Eupatilin; (**D**), Cineol; (**E**), a-Terpineol; (**F**), Borneol; (**G**), Camphor; (**H**), L-Carveol or (**I**), Thujone.

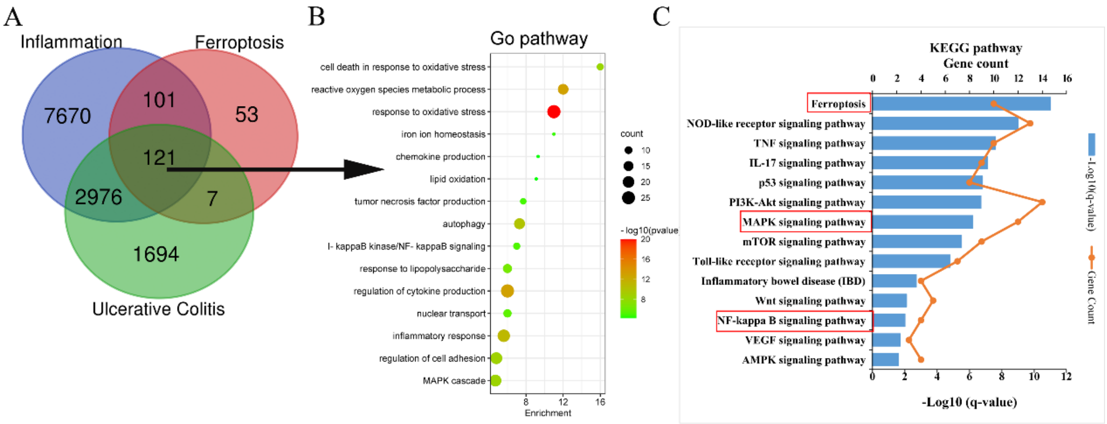

Figure S2. Functional enrichment analysis of inflammation, ulcerative colitis and ferroptosis. (A), A total of 10,868 inflammation-related genes, 282 ferroptosis-related genes and 4,798 UC-related genes were identified using the GeneCards database. (**B&C**), GO and KEGG functional enrichment analysis of common genes among inflammation, ulcerative colitis and ferroptosis.

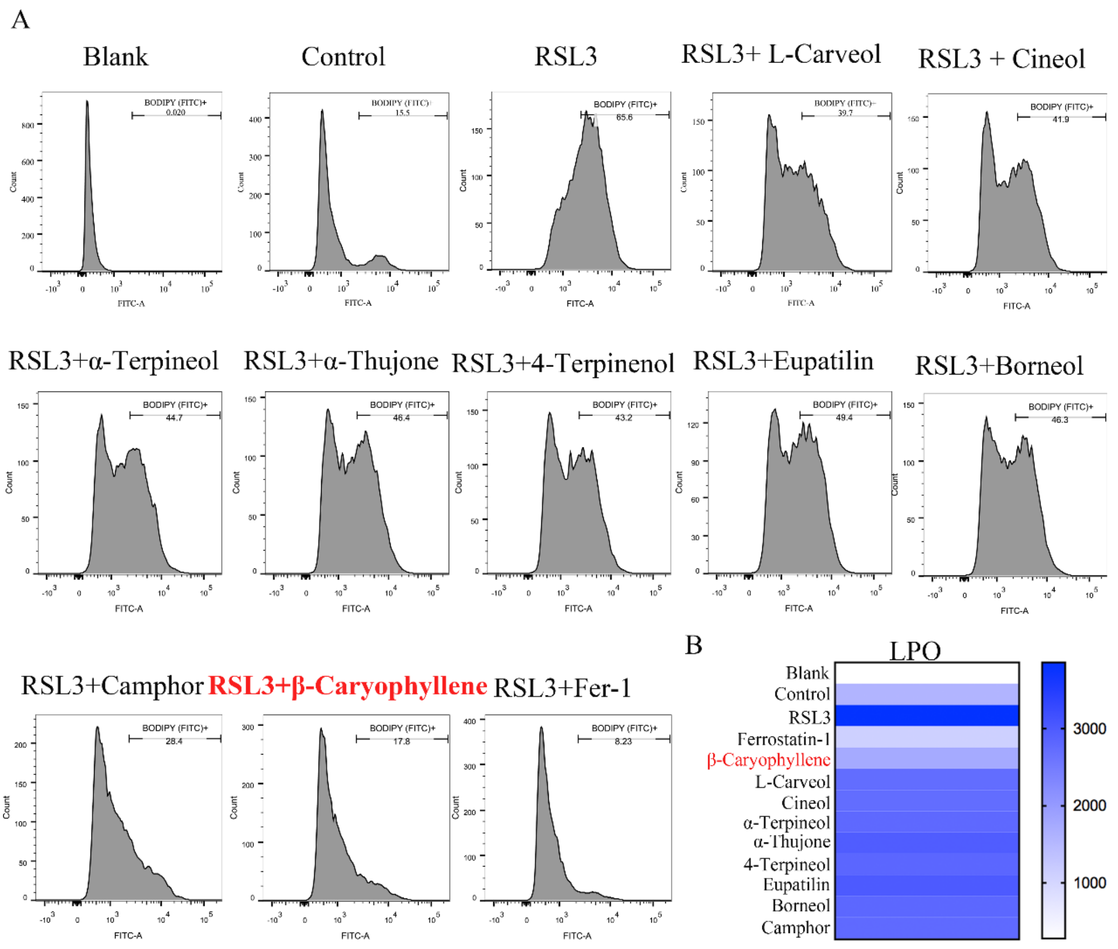

Figure S3. Screening of main components of essential oil from Artemisia argyi leaf against lipid peroxidation. (A&B), Representative flow cytometry images, percentage of lipid ROS and fluorescence intensity showing lipid peroxidation after different treatments.

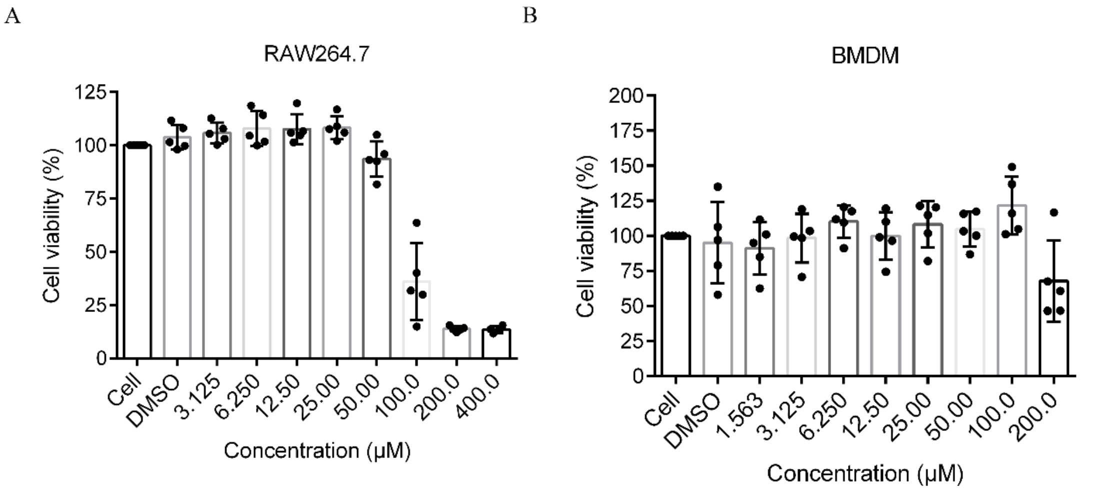

Figure S4. Effects of BCP on cell viability of RAW 264.7 macrophages and BMDMs. Data are representative of five independent experiments.

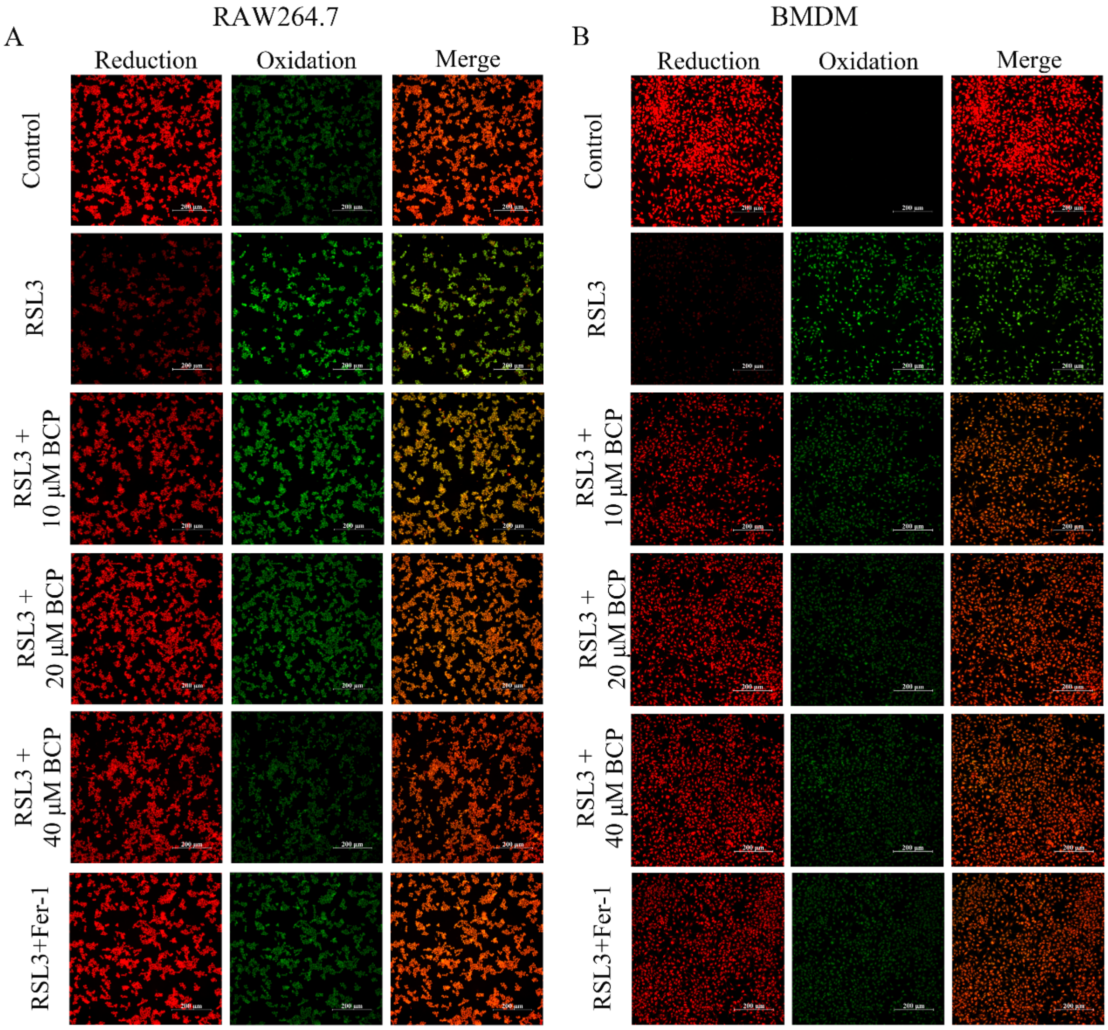

Figure S5. BCP exerts an inhibitory effect on RSL3-induced lipid peroxidation. RAW264.7 macrophages or BMDMs were treated with 500 nM RSL3 in the presence of different concentrations of BCP or 400 nM Fer-1 for 6 h. (**A&B**), Representative C11-BODIPY staining images showing lipid peroxidation of RAW264.7 macrophages and BMDMs. Scale bar, 200 μm. Data are representative of three independent experiments.

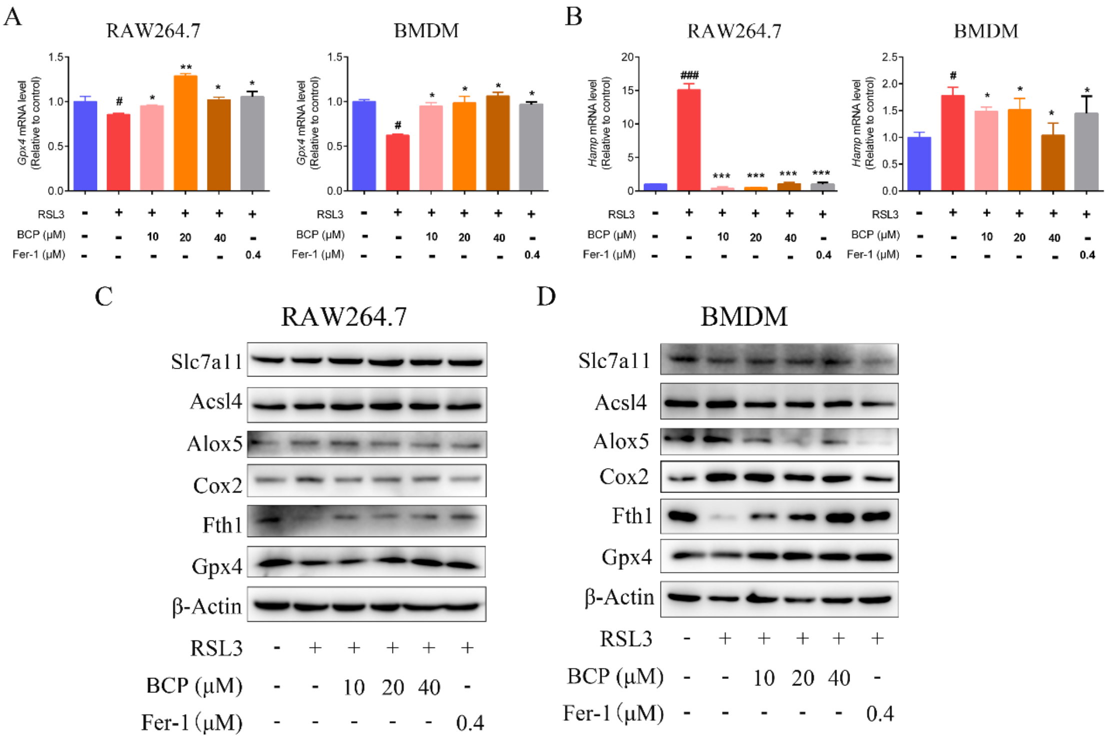

Figure S6. BCP regulates the expression of ferroptosis-related genes and proteins. RAW264.7 macrophages or BMDMs were treated with 500 nM RSL3 in the presence of different concentrations of BCP or 400 nM Fer-1 for 6 h. (**A&B**), Relative mRNA levels of Gpx4 and Hamp in RAW264.7 macrophages and BMDMs were detected by qRT-PCR. Data are presented as mean ±S.E.M. using Gapdh as a reference. #$p < 0.05$, ###$p < 0.001$ versus control group; *$p < 0.05$, **$p < 0.01$, ***$p < 0.001$ versus RSL3 treatment group. (**C&D**), Relevant protein levels of ferroptosis in RAW264.7 macrophages and BMDMs were analyzed by immunoblotting. β-Actin was used as an internal reference.

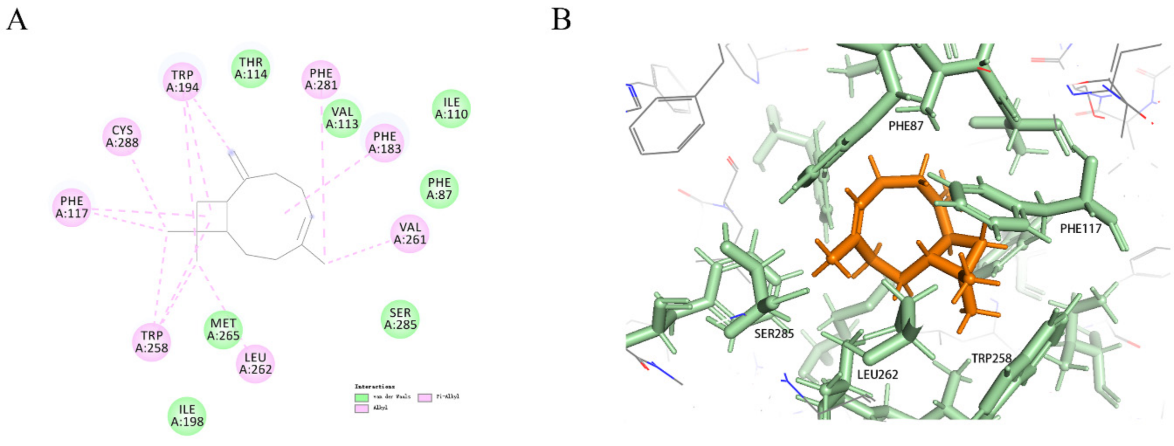

Figure S7. Molecular docking diagram of BCP binding to the CB2R. (A &B), 2D interaction plot and 3D interaction plot.

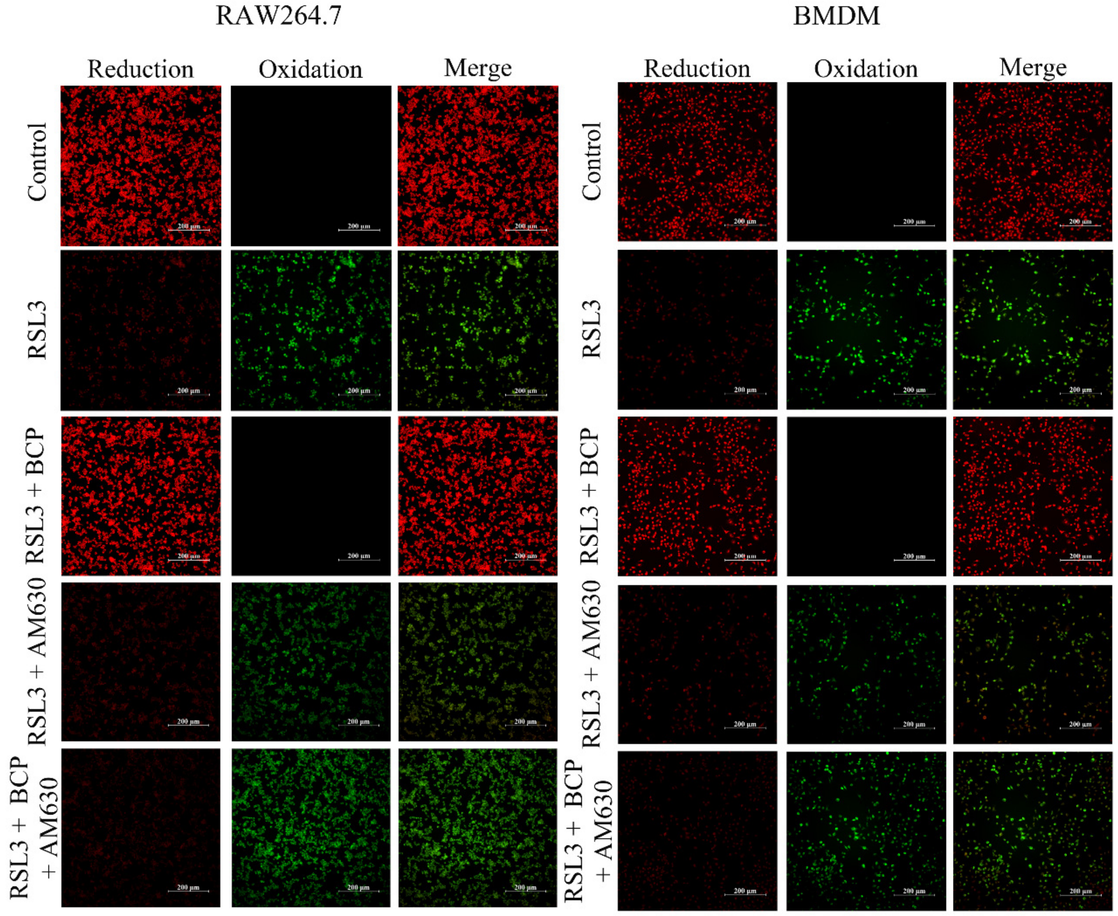

Figure S8. BCP inhibits RSL3-induced lipid peroxidation by activating the CB2R. RAW264.7 macrophages or BMDMs were treated with 500 nM RSL3 in the presence of BCP (20 μM), CB2R antagonist AM630 (5 μM) or both of AM630 and BCP for 6 h. Representative C11-BODIPY staining images showing lipid peroxidation of RAW264.7 macrophages and BMDMs. Scale bar, 200 μm. Data are representative of three independent experiments.

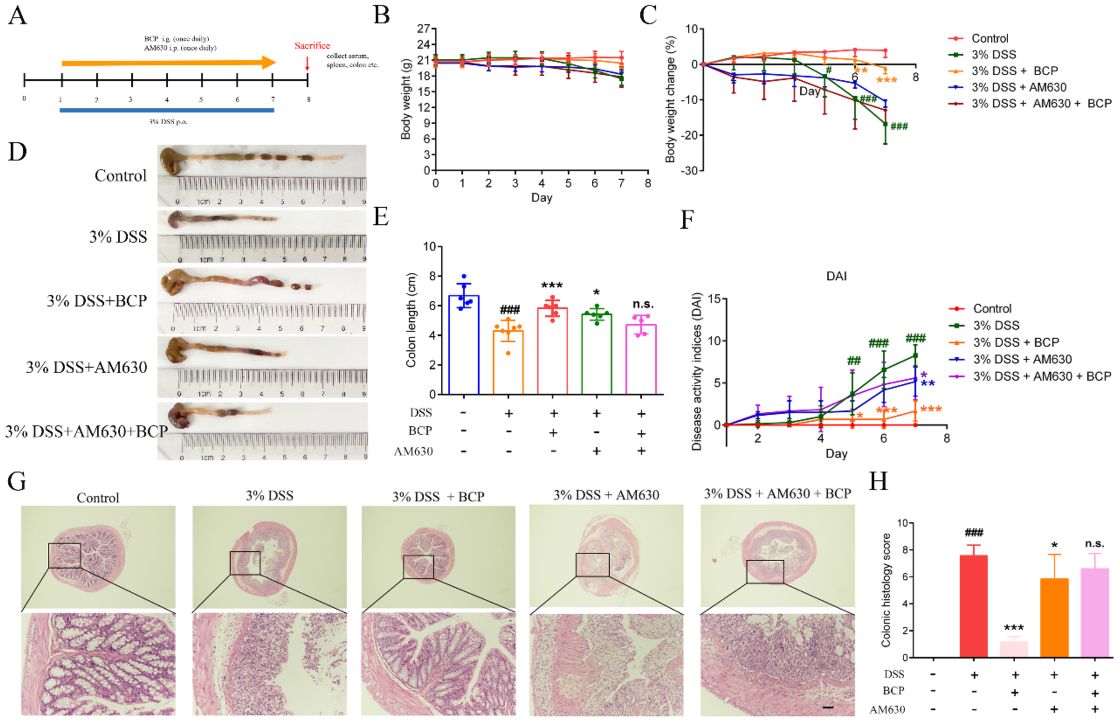

Figure S9. BCP mitigates DSS-induced ulcerative colitis via activation of the CB2R. Mice were given 3% DSS for 7 days and were treated with BCP (50 mg/kg, p.o.) alone, with the CB2R selective antagonist AM630 alone, or with AM630 (30 minutes before) plus BCP. (**A**), Diagram of the in vivo study design. B&C, Body weight and body weight changes of mice in different treatments. (**D&E**), Comparison of colon length and graphs of colon length. (**F**), DAI were determined according to scoring standards. (**G&H**), HE staining of colonic tissues and colonic histology score were analyzed after the last administration. Scale bar, 50 μm. Data are presented as the mean ± S.E.M. of 6 colonic tissues samples. Statistical analyses were performed with one-way ANOVA. #$p < 0.05$, ##$p < 0.01$, ###$p < 0.001$ versus mice from control group; *$p < 0.05$, **$p < 0.01$, ***$p < 0.001$ versus mice from 3% DSS group.

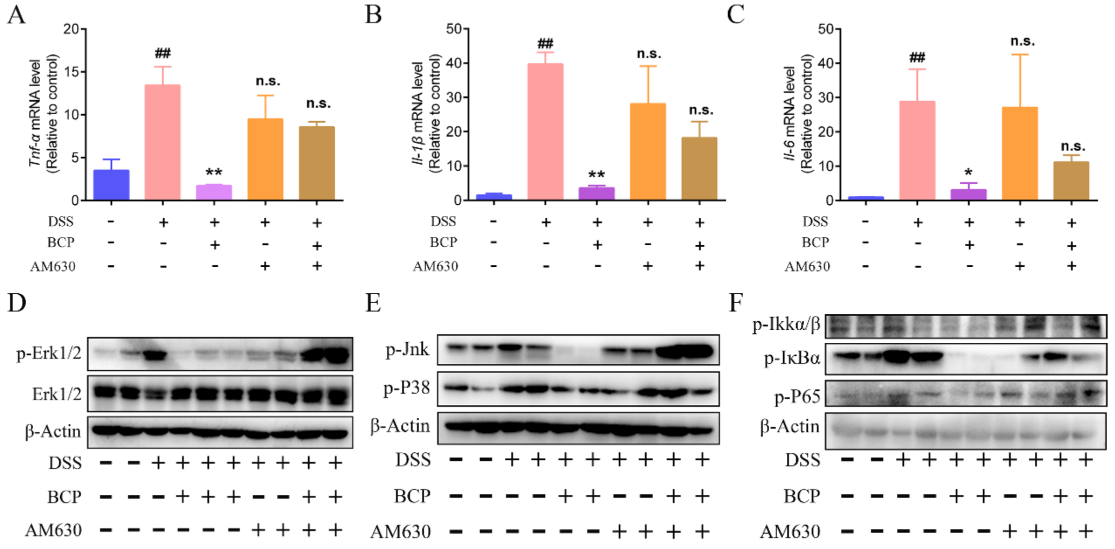

Figure S10. BCP suppresses the DSS-induced inflammatory response by activating CB2R. (A-C), Relative mRNA expression levels of Tnf-*α*,

Il-1β and Il-6 in colonic tissues were determined by RT-qPCR. Data are presented as the mean ± S.E.M. of 6 colonic tissues samples using Gapdh as a reference. (**D-F**), Levels of p-Erk1/2, Erk1/2, p-Jnk, p-P38, p-Ikkα/β, p-IκBα and p-P65 in mice with DSS-induced colitis treated with BCP or AM630 were determined by western blot analysis. β-Actin was used as an internal reference. Statistical analyses were performed with one-way ANOVA. ##p < 0.01 versus mice from control group; *p < 0.05, **p < 0.01 versus mice from 3% DSS group.
